# Characteristics that influence purchase choice for cannabis products: a systematic review

**DOI:** 10.1186/s42238-022-00117-0

**Published:** 2022-02-01

**Authors:** Jennifer Donnan, Omar Shogan, Lisa Bishop, Michelle Swab, Maisam Najafizada

**Affiliations:** 1 School of Pharmacy, Memorial University of Newfoundland and Labrador, St. John’s, Canada; 2 Faculty of Medicine, Memorial University of Newfoundland and Labrador, St. John’s, Canada

**Keywords:** Cannabis, Public health, Cannabis policy, Choice attributes, Purchase decisions, Price elasticity

## Abstract

**Introduction:**

When non-medical cannabis use became legal, government regulators implemented policies to encourage safer consumption through access to a regulated market. While this market is growing, sales still occur through unregulated channels. This systematic review identifies factors influencing cannabis purchasing to help policymakers understand why consumers still purchase illicit market cannabis (registered with PROSPERO CRD42020176079).

**Methods:**

A comprehensive search strategy included databases in health, business, and social science fields (inception to June 2020). Studies were eligible for inclusion if they were conducted with persons who purchase cannabis and examine at least one attribute that would influence purchase choice and were published in the English language. Studies could be of any methodological design. Two independent reviewers completed two levels of screening, and all extraction was verified by a second reviewer. A qualitative synthesis of the findings was completed. The quality of the included studies was assessed using the Mixed Methods Appraisal Tool.

**Results:**

Of the 4839 citations screened, 96 were eligible for full-text review and 35 were included in the final synthesis. Aspects of price were the most common factors (27 studies). Twenty studies measured price elasticity; most studies found that demand was price inelastic. Many other attributes were identified (e.g., product quality, route of administration, product recommendations, packaging), but none were explored in depth. Eleven studies addressed aspects of product quality including demand elasticity based on quality, potency, and aroma. Studies also explored consumer-perceived “quality” but provided no definition; differences in quality appeared to impact consumer choice. Smoking cannabis appeared to be the preferred route of administration but was only examined in three studies. There was insufficient data to understand in the impact of other attributes on choice. There appeared to be preference heterogeneity for different attributes based on the consumer’s experience, reason for use, and gender.

**Conclusion:**

While price influences choices, demand is relatively inelastic. This suggests that consumers may be seeking lowest-cost, unregulated cannabis to avoid reducing consumption. Beyond price, there is a significant gap in our understanding of consumer choices. Perceived quality does appear to impact choice; however, more research is needed due to the lack of a recognized definition for cannabis quality.

**Supplementary Information:**

The online version contains supplementary material available at 10.1186/s42238-022-00117-0.

## Introduction

Cannabis is the second most commonly used psychoactive substance world-wide (Peacock et al., 2018; United Nations Office on Drugs and Crime, 2019). The global estimated annual prevalence of cannabis consumers aged 15–64 was 3.8% in 2017 or approximately 188 million people (Peacock et al., 2018). The number of people who use cannabis annually is also estimated to have increased by roughly 30% between 1998 and 2017 (United Nations Office on Drugs and Crime, 2019).

This rise may be accredited to recent changes in cultural-norms and policies in several countries regarding cannabis use (Bahji and Stephenson, 2019; National Academies of Sciences et al., 2017; National Institute of Health, 2015). Currently, cannabis for non-medicinal use is legal in Canada, Georgia, South Africa, Uruguay, the Australian Capital Territory in Australia, and specific regions in the USA (ACT Government, 2020; BBC News, 2018; Guthrie, 2018; United Nations Office on Drugs and Crime, 2019). Within the USA, there are 19 states and the federal District of Columbia which have legalized recreational cannabis (Solutions, 2019). Several countries have also adopted milder forms of punishment in regulating cannabis without actual legalization, through decriminalization or unenforced laws (Areesantichai et al., 2020; Hanford, n.d.; Smith, 2020). Moreover, medicinal use of cannabis has been prevalent and legalized in many countries for some time.

Illegal cannabis sales are still largely prevalent in Canada and beyond, with only 48% of Canadian cannabis consumers making their last purchase from a legal source and illegal retailers in California outnumbering legal retailers three to one (Wadsworth et al., 2021). People who use cannabis attribute the persistence of the illegal market to numerous issues that may decrease the appeal of legal cannabis. According to the media, cannabis consumers reported issues such as high cost (Deschamps, 2020; Esfandiari, 2019; Fahmy, 2019; Johnson, Glen et al., 2019; McCabe, 2019; Shackford, 2019; The Canadian Press, 2020; Tunney, 2019a), poor cannabis quality (Ahearn, 2018; Turvill, 2020), product moisture (Israel, 2019; Turvill, 2020), limited supply (CBC News, 2019; Cecco, 2019; Esfandiari, 2019; Geraghty, 2019; Johnson, Glen et al., 2019; Mazur, 2019; Tunney, 2019a; Williams, 2019), distance to licensed stores (Esfandiari, 2019; Johnson, Glen et al., 2019; Tunney, 2019b), and inconvenient packaging (Lamers, 2019). Through crowdsourced cannabis prices, Statistics Canada confirmed that the price of legal cannabis is more expensive compared to illegal cannabis (Statistics Canada, 2020). From 2018 to 2019, the average price of legal cannabis in Canada increased from $9.69 per gram to $10.30, while the average price of illegal cannabis dropped from $6.44 per gram to $5.73 (Statistics Canada, 2020). This mirrors the experience in the USA where illegal cannabis prices dropped substantially in states where it became legalized (Smart et al., 2017).

The multi-attribute utility theory (Torrance et al., 1982) states that when individuals make decisions, their choices are based on their preferences towards certain attributes of that choice. Likewise, there are many attributes or factors that people consider when making the choice between legal or illegal cannabis. A better understanding of the degree to which these specific factors influence decisions can help inform cannabis policy. Research to date has predominantly examined the effect of cannabis price on consumer demand by measuring price elasticity of demand. Price elasticity of demand (Gilroy et al., 2020) represents the degree to which demand for cannabis changes as price fluctuates. A common method to examine price elasticity has been the marijuana-purchase task (MPT) (Aston and Meshesha, 2020). The MPT is a simulated purchase scenario which evaluates consumers’ demand for cannabis in relation to a change in price (e.g., from free to $10 over 20 increments). This method is also used to look at demand elasticity in relation to characteristics other than price, such as product quality.

There are considerations or attributes beyond price that are important to consumers when they purchase cannabis. Some research has been done to focus on factors like quality, aroma, potency, packaging, and warning labels. However, because cannabis legalization is a new in many countries, there is a lack of research that attempts to bring existing research evidence on cannabis choice behavior together. Understanding the role that all of attributes of choice play in decision making may be informative for refining cannabis policies to better support public health and safety as well as meet consumer needs. This can also offer insight for countries looking to legalize cannabis for either medicinal or non-medicinal use. The purpose of this systematic review is to identify what factors influence cannabis purchasing behavior to inform the design of a cannabis choice modeling study. The secondary objective was to identify gaps and limitations in the existing evidence base.

## Methods and analysis

### Study design

This study was designed in accordance with the PRISMA statement on systematic reviews (Moher et al., 2009) and is registered (CRD42020176079) with PROSPERO (International Prospective Register of Systematic Reviews). The Covidence online systematic review software was used to assist in screening, selection, and data extraction.

### Eligibility criteria

The research team used the SPIDER search strategy tool (Cooke et al., 2012) to define the key elements of the review question. This tool is designed specifically for research questions that lend themselves better to qualitative or mixed methods approaches. The inclusion and exclusion criteria along with the SPIDER search protocol are described in Table 1.

**Table 1:** Study eligibility criteria

*Sample:* individuals who have consumed cannabis for either medical or non-medical purposes	
*Phenomenon of interest:* consumer choice for cannabis products (could be either legal or illegal sources; and for either medicinal or non-medicinal purposes)	
*Design:* any study design, including but not limited to focus groups, interviews, case studies, observational studies or surveys were included. Non-English articles, systematic or literature reviews were excluded. Studies where only abstracts were available were also excluded.	
*Evaluation:* at least one situational attribute of choice (e.g., product characteristics, retailer characteristic)	
*Research type:* qualitative, quantitative or mixed methods	

### Data sources and search strategy

A comprehensive search strategy which aimed to find both published and unpublished studies was developed in conjunction with an experienced librarian (MS) and peer reviewed by a second librarian. The search included databases in health (Medline, EMBASE, PsycINFO), business (ABI/INFORM, Business Source Complete), and social science (ASSIA, IBSS, SocINDEX, Sociological Abstracts) fields. A broad index search in Scopus was also performed. The complete search strategy is included in the appendix. The strategy was first created in Ovid MEDLINE and was modified to fit other databases’ search criteria. Reference lists of key articles were also screened (JD). The search was conducted from inception to June 2020 to each database.

### Screening and selection process

Two reviewers (JD, OS) independently screened articles in Covidence utilizing a two-stage screening process based on the eligibility criteria. In the first stage, articles were screened based on the title and abstract. Articles which did not meet the inclusion criteria were excluded. Disagreements were subsequently resolved via discussion until consensus was achieved. In the second stage, full-text screening of the included articles was independently performed by both reviewers to determine eligibility. Reasons for exclusion during this stage were documented. Disagreements were again resolved through discussion. A third reviewer (LB) was consulted in select cases when meeting the inclusion criteria was unclear.

### Data extraction

Extracted information included study characteristics, participant characteristics, and attribute characteristics. Study characteristics included year of publication, methods used, country, time period of data collection, and sample size. Participant characteristics included gender/sex and non-medicinal vs. medicinal vs. dual use (both non-medicinal and medicinal) use of cannabis. Attribute characteristics deemed to be relevant to consumer choice, as well as a narrative summary of these characteristics, were also extracted.

### Data synthesis

Because these attributes were investigated using a diverse range of methodologies and recruited a variety of study populations, there was no attempt to combine studies statistically. Only qualitative synthesis was completed. Where possible, data exploring differences in preferences among sub-groups of the population or between the legal and illegal market were highlighted.

### Quality assessment

The Mixed Methods Appraisal Tool (MMAT) (Hong et al., 2018) was used to assess the quality of each of the included studies. The MMAT tool provides five critical appraisal questions for each of the five possible study design categories. Only two categories were required for appraisal in this systematic review. These were “qualitative” and “quantitative descriptive.” For qualitative studies, questions cover appropriate method, findings adequately derived from the data, interpretation substantiated by data, and coherence between qualitative sources. For quantitative descriptive studies, questions cover: the appropriateness of sampling, representativeness of sample, appropriate measure, overall risk of bias being low, and appropriateness of statistical analysis. Each question has three possible responses: yes, no, and cannot tell. Quality assessment was completed by two reviewers (JD, OS), and disagreements were resolved through discussion. Studies were not excluded based on not meeting a quality threshold, but rather quality assessment was considered in the interpretation of the findings.

## Results

**Fig. 1:**
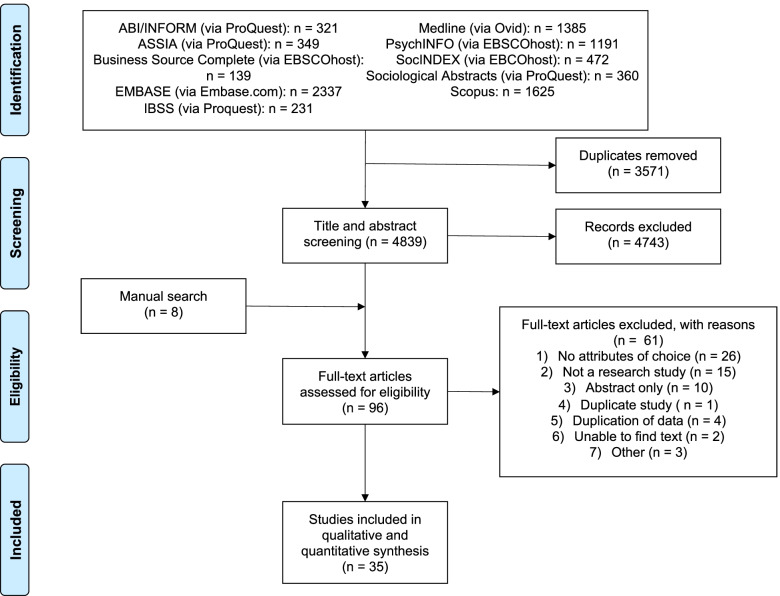
PRSIMA flow diagram of studies’ screening and selection

**Fig. 2:**
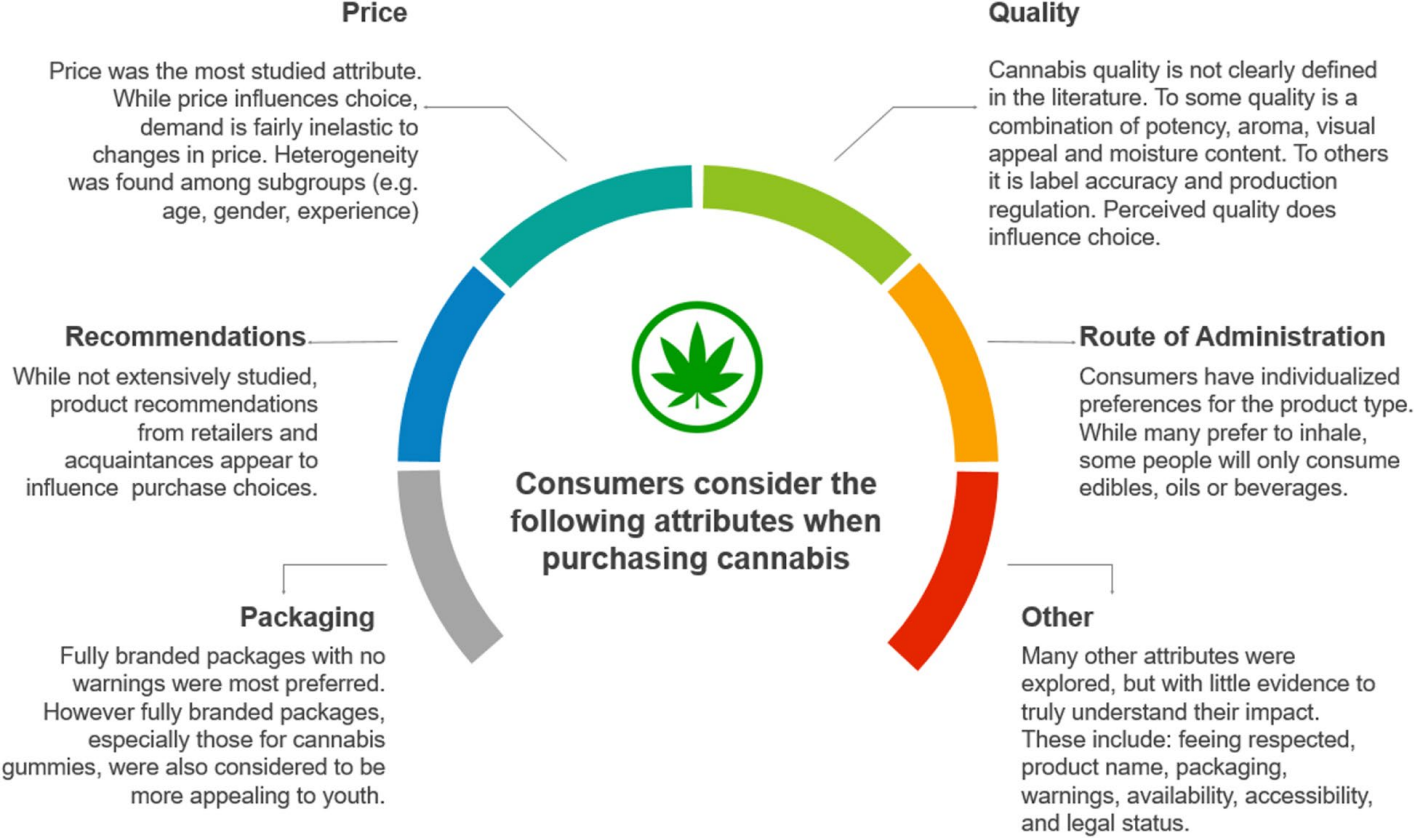
Emerging themes on attributes of choice for cannabis products

A total of 4839 titles and abstracts were screened after duplicates were removed. Ninety-six articles were eligible for full-text review; of these, 61 were excluded due to (1) no attributes of choice (*n* = 26), (2) not a research study (*n* = 15), (3) abstract only (*n* = 10), (4) duplicate study (*n* = 1), (5) duplication of data (*n* = 4), (6) unable to find text (*n* = 2), and other (*n* = 3). A total of 35 publications were included (Fig. 1, Table 2). Most were conducted within the USA (*n* = 25); five were carried out in Canada, six in other international locations, and one was of unknown location (three studies were conducted in more than one country). The most frequently examined attribute was price, with twenty-seven studies looking at some measure of the impact of price on choice. Most studies were conducted in a population where cannabis was not legalized for non-medical use (*n* = 19), some were conducted in legalized environments (*n* = 9), while other had unknown or mixed legalization status (*n* = 8). Only fourteen studies (Aston et al., 2019; Boehnke et al., 2019; Capler et al., 2017; Chait and Burke, 1994; Cole et al., 2008; Gilbert and DiVerdi, 2018; Goodman et al., 2019; Goudie et al., 2007; Halcoussis et al., 2017; Reinarman, 2009; Riley et al., 2020; Shi et al., 2019; Shukla, 2003; Wadsworth et al., 2019; Williams, 2004) explored non-price attributes, further categorized into the following themes: quality, route of administration, packaging, and product recommendations (Fig. 2).

**Table 2:** Description of included studies

Author, year	Method (hypothetical/revealed)*	Attributes	Population Non-medical legalization status	Sex (% female) Mean age (SD)	Time period	Location
Amlung, 2019	MPT (hypothetical)	Price elasticity Substitutability	289—adult cannabis users; Immediately before cannabis legalization	40.1% 31.7 (9.9)	November 2017 to February 2018	Hamilton, ON, Canada
Amlung, 2019	MPT (hypothetical)	Price elasticity Substitutability	724—adult cannabis users (> 21 years); States where cannabis is legalized	52% 34.13 (10.02)	September to December 2017	Alaska, California, Colorado, Massachusetts, Maine, Nevada, Oregon, Washington, USA
Aston, 2015	MPT (hypothetical)	Price elasticity	99—frequent cannabis users (18–44 years); cannabis not legalized	37.4% 21.4 (4.4)	2010–2011	Rhode Island, USA
Aston, 2016	MPT (hypothetical)	Price elasticity	83—frequent cannabis users (18–44 years); cannabis not legalized	34.9% 21.6 (4.7)	2010–2011	USA
Cole, 2008	MPT (hypothetical)	Quality elasticity	80—polydrug users 18–44 years; cannabis not legalized	Polydrug users: 36.3% 21.0 (1.2) Cannabis users: 36.3% 21.0 (1.2)	2006	England
Collins, 2014	MPT (hypothetical)	Price elasticity	59—young participants (18–25 years) who regularly used cannabis; cannabis not legalized	46% 21.64 (1.98)	Unknown	Buffalo, USA
Goudie, 2007	MPT (hypothetical)	Income elasticity Quality	40—polysubstance users. 38—reported at least a single lifetime use of cannabis; cannabis not legalized	Polysubstance users: 27.5% 23.8 (4.9 Cannabis users: unknown	2005	England
Hindocha, 2017	MPT (hypothetical)	Price elasticity	24—non-dependent cannabis and tobacco smokers (18–60 years); cannabis not legalized	50% 24.46 (3.96)	Unknown	London, UK
Nisbet, 1972	MPT (hypothetical)	Price elasticity, expenditure elasticity	926 UCLA students (437 users of cannabis); cannabis not legalized	Unknown	Unknown	Los Angeles, California, USA
Patel, 2019	MPT (hypothetical)	Price elasticity	749—adults with cannabis use in the previous 6 months; states where cannabis is legalized	53.2% 37.7 (10.2)	September to December 2017	USA
Peters, 2017	MPT (hypothetical)	Price elasticity	82—frequent cannabis users, 18 years or older (use 20+ days in past month); unknown legalization status	54.9% 32.4 (8.8)	Unknown	USA
Strickland, 2017	MPT (hypothetical)	Price elasticity	78—frequent cannabis users, who consumed at least once in the previous 2 weeks, and at least 50 lifetime uses; unknown legalization status	51.6% 30.2 (7.3)	Unknown	USA
Strickland, 2019	MPT (hypothetical)	Price elasticity	83—non-medical prescription opioid users > 18 years of age; unknown legalization status	63.9% 34.0 (8.0) Cannabis users: unknown	May to September 2018	USA
Teeters, 2019	MPT (hypothetical)	Price elasticity	132—college students who reported use on 4 or more days in past month; unknown legalization status	46.2% 19.94 (3.23)	2014–2016	USA
Vincent, 2017	MPT (hypothetical)	Price elasticity	683—young adult (18–25 years) non-medicinal cannabis users; unknown legalization status	16% 21.2 (2.2)	Unknown	USA
Aston, 2019	Qualitative interviews (revealed)	Route of administration	25—medical cannabis registration card holders; cannabis not legalized	60% 47 (12)	2016	Rhode Island, USA
Ben Lakhdar, 2016	Secondary analysis of data from the French National Identification System for Drugs and Other Substances (SINTES) and TREND system (both surveys) (Revealed)	Price elasticity, quantity discount	268 cannabis users (249 for the elasticity calculation); cannabis not legalized	23.1% 27 (7.234)	2005	Mainland France (Lyon, Marseille, Metz, Paris, Rennes, Toulouse)
Caulkins, 2006	Secondary analysis of survey data from the National Household Survey on Drug Abuse (revealed)	Price discounts	National probability sample of the civilian noninstitutionalized population (> 12 years of age). 8339 respondents reported using cannabis in the past 12 months; cannabis not legalized	Unavailable	2001	USA
Davis, 2016	Secondary analysis Crowdsource platform PriceOfWeed.com (revealed)	Price elasticity	23,000 actual cannabis transactions where price, quantity, and quality are reported; mixed legalization status	N/A	Between September 2, 2010, and August 29, 2011	USA (excluding Alaska and Hawaii)
Desimone, 2003	Secondary analysis of data from the National Household Surveys on Drug Abuse (revealed)	Price elasticity	92,784 individuals aged 18 to 39 43,147 individuals aged 12–17; cannabis not legalized	Individuals aged 18–39: 50.9% 29.05 (6.28) Individuals aged 12–17: 48.8% 14.49 (1.68)	1990–1997	USA
Halcoussis, 2017	Secondary analysis of crowdsourced data on prices (PriceOfWeed.com) (revealed)	Price elasticity, quality	29,461 transactions; mixed legalization status	N/A	September 2010 through March 2012	USA
Hansen, 2017	Administrative data Washington State Liquor and Cannabis Board (revealed)	Tax reform (i.e., price)	Cannabis legalized	N/A		Washington, USA
Reed, 2020	Qualitative interviews (revealed)	Price, source	60 individuals who consumed cannabis; cannabis legalized	64% 21.8 (2.53)	2014–2016	California, USA
Reinarman, 2009	Survey and Interviews in Amsterdam and San Francisco (revealed)	Source, price, potency, accessibility	Experienced users (at least 25 occasions in their life); cannabis not legalized (California), Decriminalized (Amsterdam)	Amsterdam: 41% 34.2 (standard deviation not reported) San Francisco: 47% 37.1 (standard deviation not reported)	Amsterdam 1995/1996 San Francisco 1997	Amsterdam (216), San Francisco (266)
Riley, 2020	Cross-sectional survey (revealed)	Price elasticity, quantity discount, quality	1961 cannabis consumers; cannabis not legalized	27.2% Mean age not reported	August and September 2017	South Africa
Smart, 2017	Secondary analysis of administrative data from Washington State’s cannabis traceability system	Price elasticity, potency on price, quantity discount	A total of 44,482,176 million cannabis purchases, including 31,052,123 cannabis flower purchases; cannabis legalized	N/A	July 2014–September 2016	USA (Washington)
Wadsworth, 2019	Survey - International Cannabis Policy Study (ICPS) (revealed)	Quantity discount, source	1227 Canadians aged 16–65 years who reported purchasing dried cannabis in the past 12 months; immediately before cannabis legalization	Unweighted sample: 48.8% Mean age not reported Weighted sample: 39.8% Mean age not reported	August–October 2018	Canada
Boehnke, 2019	Survey (revealed)	Cannabinoid content, cannabis strain, potency, administration routes, dispensary/friend recommendation, smell, visual properties, described effects, name	Medical cannabis patients (≥ 18 years) with chronic pain (1321) 715—medicinal only 606—used both non-medicinally and medicinally (dual); in states with cannabis legalization	781, 59% 49.8 (13.9)	January and August 2018	USA and Canada
Capler, 2017	Secondary analysis of survey data Cannabis Access for Medical Purposes Study (revealed)	Quality, safety, availability, efficiency, cost, feeling respected, product type	445 Adult medical cannabis users (215 accessed from dispensaries; 230 from other sources); cannabis legalized for medical purposes	33% (dispensary 32%; non-dispensary 34%) 39.3 (dispensary 45.5 Non-dispensary 36.3)	2011–2012	Canada
Chait, 1994	Choice blinded trials (two independent choice trial—with low (0.63% THC) and high (1.95% THC) potency cannabis) (revealed)	Potency	12 volunteers judged to be healthy with no history of substance use disorder; cannabis not legalized	25% 23 (standard deviation not reported)	Unknown	Chicago, USA
Gilbert, 2018	Rating of 13 samples using standard sensory evaluation techniques with untrained consumers (revealed)	Aroma	61 adults 21 years of age or older (current, former and non-users) Female: 46% (29) Age: 28.2 (8.4); cannabis is legalized	45.9% 28.2 (8.4)	Unknown	Colorado, USA
Goodman, 2019	An experimental choice task Part of the online International Cannabis Policy Study (hypothetical)	Packaging, warnings	Participants aged 16–65 from Canada (*n* = 9987) and US states with “legal” (*n* = 7376) and “illegal” (*n* = 9682); Canada pre-cannabis legalization, USA a mix of legal and illegal states	61.5% 44.5 (15.5)	August–October 2018	Canada and USA
Shi, 2019	Online cross-sectional survey with discrete choice experiment Survey performed (hypothetical)	THC concentration, CBD concentration, warning message, price (WTP)	2345 adults aged 21 and over (1186 past-year nonusers and 1159 past-year users); cannabis legalized in included states	Past-year nonusers: 67.96% Mean age and standard deviation not reported Past-year users: 68.51% Mean age and standard deviation not reported	October 2017	USA (California, Colorado, Massachusetts, Nevada, Oregon, and Washington)
Shukla, 2003	Key informant interviews (revealed)	Illegal market considerations, availability, cost	51—purposeful sampling of individuals who made choice about cannabis use; cannabis not legalized	47% 31.52 (standard deviation not reported)	Unknown	USA
Williams, 2004	Australian National Drug Strategy’s Household Surveys (NDSHS) (revealed)	Price, price elasticity Legal status	5468 observations from non-institutionalized population aged 14 years and older; cannabis not legalized (one region with decriminalized status)	54% Mean age and standard deviation not reported	1988–1998	Australia

*MPT* Marijuana Purchase Task; *U/K* unknown

*Some studies collect data on preferences uncovered through posing hypothetical questions/examples (hypothetical). Other studies ask participants directly their preferences and actual choices (revealed)

### Quality appraisal

Generally, studies were of appropriate quality to address the relevant study question. The design elements that were most difficult to assess were “representativeness of the sample” and “low risk of non-response.” Studies which collected data through crowdsourcing (e.g., Amazon Mechanical Turk) or participant self-identification, compared to administrative data or national surveys, are more likely to be subject to selection bias, non-response bias, and recall bias. In studies where sampling was appropriate and representative of the population, it is important to note that several studies had very narrow inclusion criteria, and therefore, the samples are only representative of that particular subset of the population. All but three studies used a quantitative methodology and were assessed using the “quantitative descriptive” set of questions. There was a wide range of methodologies used, some with more complex analyses; however, measures and statistics were generally appropriate for the specific methods used. A complete table outlining the results of the quality appraisal can be found in the online appendix.

### Price-related factors

Studies which examined price can be further divided into two categories: those that utilized a MPT design (*n* = 12) and those that did not (*n* = 15).

#### Marijuana purchase task (MPT) studies

Twelve studies used the MPT approach to examine purchase demand in relation to price, which allows for the estimation of several demand predictors (Table 3) (Gilroy et al., 2020). These include: price elasticity, which is the sensitivity of quantity purchased to increases in prices; *P*_max_, which is the price at which demand become elastic; intensity (*Q*_0_), which is the amount consumed when price is free; *O*_max_, which is the maximum expenditure; and breakpoint, which is the cost at which consumption is suppressed to zero.

**Table 3:** Summary of findings from the studies that use a Marijuana Purchase Task

Authors—year	Number of prices	Prices (per unit)	Cannabis unit	Demand equation	Significant demand predictors	Summary of results
**Price elasticity**						
Amlung and MacKillop—2019	20	free, $1, $2, $4, $6, $8, $10, $12, $14, $16, $18, $20, $25, $30, $35, $40, $45, $50, $55, and $60	Dried flower (grams)	Not reported	Intensity Price elasticity *P*_max_	- **Illegal cannabis:** elasticity = 0.0042 (alone), 0.0095 (with legal alternative); *P*_max_ = 14.09 (alone), 8.22 (with legal alternative); intensity *Q*_0=_ 9.11 (alone) - **Legal cannabis**: elasticity = 0.0029 (alone), 0.0046 (with illegal as alternative); *P*_max_ = 16.28 (alone), 9.65 (with illegal as alternative); intensity *Q*_*0*=_ 11.20 (alone) - Both are inelastic, but illegal cannabis is more elastic - Having legal cannabis as an alternative had a greater effect on the elasticity of illegal cannabis than vice versa (threefold difference). - Sensitivity analyses revealed that the asymmetric substitution pattern for legal over illegal cannabis was identical across genders, age, and income demographics
Amlung et al.—2019.	20	Free, $1, $2, $4, $6, $8, $10, $12, $14, $16, $18, $20, $25, $30, $35, $40, $45, $50, $55, $60	Dried flower (grams)	Nonlinear exponential demand curve model—(Hursh and Silberberg (2008) Exponential cross price elasticity model (Hursh, 2014)	Intensity *P*_max_ Price elasticity Substitutability	- **Illegal cannabis:** elasticity = 0.0028 (alone), 0.0047 (with legal alternative); *P*_max_ = 9.41 (alone), 6.16 (with legal alternative); intensity *Q*_0=_ 11.01 (alone) - **Legal cannabis**: elasticity = 0.0016 (alone), 0.0018 (with illegal alternative); *P*_max_ = 11.67 (alone), 10.74 (with illegal alternative); intensity *Q*_0=_15.55 (alone) - Both are inelastic, but illegal cannabis is more elastic, showing greater price sensitivity for illegal cannabis - **Substitution:** indicated as present with both fixed-price alternatives having significant positive linear cross-price elasticities (slope of illegal alternative significantly > legal alternative) - All demand indices demonstrated asymmetrical substitutability with the presence of the legal alternative increasing the elasticity of illegal cannabis to a greater degree than the reverse
Aston et al.—2015	22	$0, $0.25, $0.50, $0.75, $1, $1.25, $1.50, $1.75, $2, $2.50, $3, $3.50, $4, $4.50, $5, $5.50, $6, $6.50, $7, $8, $9, $10	Average quality hit of cannabis (assume 10 hits of cannabis in a joint; 1 joint = 1/32nd of an ounce = 0.9 g)	Nonlinear exponential demand curve model—Hursh and Silberberg (2008)	Intensity *O*_max_ *P*_max_ Breakpoint Price elasticity	- **Intensity** ***Q***_**0**_**:** 23.71 - ***O***_**max**_**:** 16.13 - ***P***_**max**_**:** 2.32 - **Breakpoint:** 4.24 - **Elasticity:** 0.04 - Income was not associated with demand
Aston et.al—2016	22	$0, $0.25, $0.50, $0.75, $1, $1.25, $1.50, $1.75, $2, $2.50, $3, $3.50, $4, $4.50, $5, $5.50, $6, $6.50, $7, $8, $9, $10	Average quality hit of cannabis (assume 10 hits of cannabis in a joint; 1 joint = 1/32nd of an ounce = 0.9 g)	Nonlinear exponential demand curve model—Hursh and Silberberg (2008)	Intensity *O*_max_ *P*_max_ Breakpoint Price elasticity	- **Intensity** ***Q***_**0**_**:** 24.94 - ***O***_**max**_**:** 16.03 - ***P***_**max**_**:** 2.31 - **Breakpoint:** 4.27 - **Elasticity:** 0.05
Collins et al.—2014	16	$0/free, 10¢, 25¢, 50¢, $1, $2, $4, $5, $7.50, $10, $15, $20, $30, $40, $80, and $160	Average-sized joint of high-grade cannabis	Modified version of the non-linear mixed effects model proposed by Hursh et al. (1998)	Intensity Breakpoint Price elasticity	- **Intensity** = ~ 10 joints when price was free - ***O***_**max**_**:** 46.63 - ***P***_**max**_**:** 13.21 - **Breakpoint** = $38.07 - **Elasticity** = − 1.75 (elastic); demand inelastic across low prices $0/free to $13/joint, but elastic for higher prices of $15 to $160/joint
Hindocha et al.—2017	23	£0, 1p,2p, 5p, 10p, 15p, 20p, 30p, 40p, 50p, 75p, £1, £1.50, £2, £2.50, £3, £3.50, £5, £5, £7.5, £10, £15, £20	Puff of cannabis	Exponentiated demand equation (Koffarnus et al. 2015)	Intensity *O*_max_ *P*_max_ Breakpoint Price elasticity	- **Intensity** ***Q***_**0**_**:** 17.14 - ***O***_**max**_**:** 652.95 - ***P***_**max**_**:** 92.19 - **Breakpoint:** 145.29 - **Elasticity:** 0.61 (when compared to placebo, cannabis was more sensitive to price)
Nisbet and Vakil—1972	Unknown	Unknown	Lids (ounces) of dry flower	Double log function	Price elasticity Expenditure elasticity	- **Price elasticity =** − 0.365 - **Expenditure elasticity =** − 0.311
Patel et al.—2019	20	$0–$60 (specific prices not reported)	Dried Flower (grams)	Nonlinear exponential demand curve model— Hursh and Silberberg (2008)	Intensity *O*_max_ *P*_max_ Breakpoint Elasticity	- **Non-DACU intensity** ***Q***_**0**_**:** 8.51 - **Non-DACU** ***O***_**max**_**:** 54.80 - **Non-DACU** ***P***_**max**_**:** 15.22 - **Non-DACU breakpoint:** 22.24 - **Non-DACU elasticity:** 0.004 - **DACU intensity** ***Q***_**0**_**:** 13.81 - **DACU** ***O***_**max**_**:** 98.92 - **DACU** ***P***_**max**_**:** 16.65 - **DACU breakpoint:** 29.83 - **DACU elasticity:** 0.002 **Note:** This study compared demand for individual who reported Driving after cannabis use (DACU) and those who did not
Peters et al.—2017	9	$0.01, $0.03, $0.10, $0.30, $1.00, $3.00, $10.00, $30.00, $1000.00	Puff of cannabis	Nonlinear exponential demand curve model—Hursh and Silberberg (2008)	Price elasticity	- **Price elasticity:** 0.0044 (95% Cl 0.0038, 0.0049) - Price elasticity did not change by gender, but was slightly different based on nicotine dependence. Both groups still showed inelastic behavior
Strickland, et al. -2017	13	$0–$11 (specific prices not reported)	Hits of cannabis (hits—10 hits/joint with 1 joint equal to 0.9 g of cannabis)	Exponentiated demand equation (Koffarnus et al. 2015)	Intensity Elasticity	- **Intensity** ***Q***_**0**_**:** 35.6 - **Elasticity:** 0.028
Strickland et al.—2019	17	$0.00 (free), $0.25, $0.50, $1, $1.50, $2, $2.50, $3, $4, $5, $6, $7, $8, $9, $10, $15, $20	Hits of cannabis (hits—10 hits/joint with 1 joint equal to 0.9 g of cannabis)	Exponentiated demand equation (Koffarnus et al. 2015)	Intensity *O*_max_ *P*_max_ Breakpoint Elasticity	- **Intensity** ***Q***_**0**_**:** 37.15 - ***O***_**max**_**:** 16.22 - ***P***_**max**_**:** 1.55 - **Breakpoint:** 3.98 - **Elasticity:** 0.007 *Note: the paper presented log transformed values, these have been reverted back for easier comparison
Teeters et al. 2019	20	$0.00—$10.00 (specific prices not reported)	Hit of cannabis (10 hits of cannabis in a joint with 1 joint equaling to 1/32 of an ounce or 0.9 g)	Exponentiated demand equation (Koffarnus et al. 2015)	Intensity *O*_max_ *P*_max_ Breakpoint Price elasticity	- **Intensity** ***Q***_**0**_**:** 24.41 - ***O***_**max**_**:** 11.93 - ***P***_**max**_**:** 1.59 - **Breakpoint:** 3.31 - **Elasticity:** 0.06
Vincent et al.—2017	9	Free ($0), $2.50, $5.00, $7.50, $10, $12.50, $15, $17.50, and $20	Low-grade, medium-grade, and high-grade joints (an average sized joint was defined as approximately 0.5 g, 5 bong hits, or 10 puffs)	Nonlinear mixed effects modeling (Pinheiro and Bates, 2000)	Intensity *O*_max_ *P*_max_ Breakpoint Price elasticity	- **Intensity** ***Q***_**0**_ **(derived):** low-grade = 4.56; medium-grade = 5.06; high-grade = 5.85 - ***O***_**max**_ **(derived):** low-grade = 8.53; medium-grade = 13.57; high-grade = 19.49 - ***P***_**max**_ **(derived):** low-grade = 5.08; medium-grade = 7.28; high-grade = 8.99 - **Breakpoint:** low-grade = 7.17; medium-grade = 9.86; high-grade = 13.10 - **Elasticity:** low-grade = −1.97; medium-grade = −1.37; high-grade = −1.11 (when the log transformation is reversed, elasticity values are 0.011, 0.043, and 0.078 respectively) - **Note:** Values in this study were square-root or log transformed.
**Quality elasticity**						
Cole et al.—2008	Cash on hand/income—fixed at 1 level	£40	*All dry flower*: poor, average, and good quality. All £15 per 1/8 oz. (3.5 g).	Not specified	Quality Elasticity	- **Quality elasticity:** − 1.31 - There were significant correlations between the self-reported number of cannabis joints used per episode and purchases of cannabis in the average and good quality conditions, but not in the poor-quality condition - As quality of cannabis decreased so did purchases for average and poor quality cannabis compared to good per individual. The number of individuals purchasing cannabis also decreased
Goudie et al.—2007	Cash on hand/income—8 levels	£20, £25, £30, £35, £40, £45, £50, £55	*All dry flower*: poor quality: £10 per 1/8 oz. Average quality: £15 per 1/8 oz. Good quality £20 per 1/8 oz.	Not specified	Income elasticity over different levels of quality	- **Income elasticity:** poor quality (− 0.21); average quality (1.16), good quality (3.14) over all income levels - Significant interaction between quality and income for the number of units purchased - Number of respondents purchasing at least a single unit of cannabis at each income level increased significantly for good quality cannabis.

Elasticity, sensitivity of consumption to increases in prices; *P*_max_, the price at which demand become elastic; intensity (*Q*_0_), the amount consumed when price is free; *O*_max_, maximum expenditure; breakpoint, cost at which consumption is suppressed to zero

Elasticity (*α*), in the context of purchase task studies, refers to the rate that point elasticity changes as a function of price. Generally, elasticity values for included studies were small (*α* < 0.01) (Amlung et al., 2019; Amlung and MacKillop, 2019; Aston et al., 2015, 2016; Collins et al., 2014; Hindocha et al., 2017; Nisbet and Vakil, 1972; Patel and Amlung, 2019; Peters et al., 2017; Strickland et al., 2017, 2019; Teeters et al., 2019). Amlung and MacKillop (2019) and Amlung et al. (2019) compared elasticities of illegal and legal cannabis products. While both were inelastic, illegal cannabis was more elastic than legal. Collins et al. (2014) used a much wider price range than other studies with price per joint ranging from $0–$160. Therefore, they found the demand was elastic at the mean; however, demand was inelastic at the lower range (up to $15) and changed to elastic at the higher ranges (over $15).

Two studies examined to what extent legal and illegal cannabis were substitutes for one another (Amlung et al., 2019; Amlung and MacKillop, 2019). These studies found when legal cannabis was available, illegal cannabis became more elastic and demand more responsive to changes in price in the illegally sourced product. The presence of illegal cannabis did not have a significant impact of the demand elasticity of legal product. Substitutability was also demonstrated, as the maximum expenditure for illegal cannabis (*P*_max_) was much lower in the presence of legal cannabis. However, the maximum expenditure on legal cannabis did not drop to the same extent in the presence of illegal cannabis.

#### Non-marijuana purchase task (non-MPT) studies

There were fifteen non-MPT studies which examined aspects of price (Table 4), including price elasticity of demand (*n* = 8) (Ben Lakhdar et al., 2016; Davis et al., 2016; Desimone and Farrelly, 2003; Halcoussis et al., 2017; Hansen et al., 2017; Reinarman, 2009; Riley et al., 2020; Williams, 2004), quantity discount (*n* = 5) (Ben Lakhdar et al., 2016; Caulkins and Pacula, 2006; Riley et al., 2020; Smart et al., 2017; Wadsworth et al., 2019), relative importance of price (*n* = 1) (Shi et al., 2019), and price by source (*n* = 3) (Capler et al., 2017; Wadsworth et al., 2019).

**Table 4:** Summary of findings from non-Marijuana Purchase Task studies

Reference	Method	Category	Summary of findings	
Aston, 2019	Qualitative interviews	Route of administration	**Route:** Vaporizing was noted to be a common method of consumption for medical users. Reasons identified for this preferred route included flexibility in dosing and timing, and the device being portable and discreet	
Ben Lakhdar, 2016	Secondary analysis if survey data	Price	**Quantity discount:** Significant negative correlation between price/gram and quantity purchased. Larger cities had lower prices/gram. Potency (real or perceived) had little impact on discount **Price elasticity:** The short-term price point elasticity for the sample was − 2.06 (range − 1.7 to − 2.1 when controlled for individual characteristics)	
Boehnke, 2019	Primary analysis of survey data	Quality	**Potency:** THC to CBD ratio relevant to ~ 70%. Most preferred ratios were high THC to high CBD (37%) and low THC to high CBD (33.7%). < 5% preferred low THC: low CBD, only THC, or only CBD, respectively. Female and medicinal users preferred low THC to high CBD ratios significantly more than their counterparts **Cannabis strain:** Preference for specific strain: indica/sativa blend (59.6%); indica (28.7%); and sativa (11.8%). More important to male, dual users, and experienced users **Described effects:** Relevant to 52% of the sample **Smell:** Relevant to 25.6% of sample. More important to male, dual users, and experienced users **Visual properties:** Relevant to 26.3% of the sample. More important to male, dual users, and experienced users	
		Route of administration	**Route:** Female, medicinal only, and novice users were less likely to smoke or vaporize (all *P* < .0001), but more likely to rank edibles, tinctures, and topicals as a first-choice route of administration	
		Recommendation	**Recommendation:** 54.9% relied on advice from dispensary employees, 2.6% consulted a medical professional	
		Other	**Product name:** Relevant to 14.2% of sample	
Capler, 2017	Secondary analysis if survey data	Price	**Cost:** Grower, self-produce, Health Canada, friend, dispensary, street	Note: Each parameter of quality, safety, availability, efficacy, cost, and feeling respected were rated based on the medicinal source, in order of best to worst
		Quality	**Quality:** Dispensary, grower, self-produce, friend, street, Health Canada	
		Other	**Safety:** Grower, dispensary, self-produce, Health Canada, friend, street **Availability:** Dispensary, grower, self-produce, Health Canada, friend, street **Efficiency:** Dispensary, self-produce, grower, Health Canada, friend, street **Feeling respected:** Dispensary, grower, friend, self-produce, Health Canada, street	
		Route of administration	**Route:** 59% preferred smoking; 25% preferred oral method of consumption	
Caulkins, 2006	Secondary analysis if survey data	Price	**Quantity discount:** The average price paid per gram drops as quantities purchased increases from $7.84/g for purchases < 5 g to $0.49/g for purchases > a pound	
Chait, 1994	Cannabis choice trial	Quality	**Potency:** In 21 out of 24 trials, participants chose the high potency over the low potency product	
Davis, 2016	Analysis of crowdsourced data	Price	**Price elasticity:** Price elasticity of demand estimates ranges between − 0.67 and − 0.79 (using ordinary least squares) **Price by quality:** People were paying more for higher quality cannabis with high-quality at an average of $13.77 per gram and low-quality at $5.63 a gram	
Desimone, 2003	Secondary analysis if survey data	Price	**Price point elasticity:** 18–39 years old: 0.018 for base model (− 0.287 to − 0.0292 for 5 different models accounting for different law enforcement variables); 12–17 years old: − 0.002 for the base model (− 0.169 to − 0.014 for 5 accounting for law enforcement variables)	
Gilbert, 2018	Sensory evaluation	Quality	**Aroma:** Study showed that users perceive differences among strains, and that there are strain clusters based on odor similarity. Aroma profiles were linked to perceptions of potency, price, and smoking interest The citrus cluster (citrus, lemon, sweet, and pungent) was perceived as more potent and were associated with higher interest and estimated price compared to the earthy cluster (earthy, herbal, and woody)	
Goodman, 2019	Experimental choice task	Packaging	**Packaging**: Fully branded packages were more appealing and more likely to be to considered youth oriented (*p* < 0.001) compared to plain packaging/brand logo only. Compared to pre-rolled joints and oils, packages for edible gummies were rated as more likely to be youth-oriented (*p* < 0.001). **Warnings**: Compared to no warnings, packages with health warnings were less appealing (*p* < 0.001)	
Halcoussis, 2017	Analysis of crowdsourced data	Price	**Price point elasticity:** − 0.418 (controlled for quality, total number of arrests for possession or sale of cannabis in each county, median household income in each county, and quality)	
		Quality	**Quality elasticity:** Both low quality and high quality cannabis were bought in higher quantities than medium quality, when all other variables are held constant (note: quality was self-reported)	
Hansen, 2017	Analysis of admin data	Price	**Price elasticity:** Measured over a 4-month time frame (2 months before and 2 months after a tax reform). Short-term price point elasticity = − 0.43, but closer to − 1.0 within 2 weeks of tax reform, i.e., demand is price point inelastic in the short-run, but price point elastic close to a price increase	
Reed, 2020	Qualitative interviews	Price	**Price by source:** A respondent reported shopping around based on deals for new patients	
		Other	**Delivery:** Most people interviewed preferred to visit dispensaries in person, though some valued the convenience of delivery **Source:** One person indicated they avoided intermediaries and preferred to buy directly from the source **Note:** Data reported from this study represents opinions of individuals or small numbers	
Reinarman, 2009	Primary survey analysis and interviews in Amsterdam and San Francisco	Price	**Price elasticity:** Regardless of source, most found prices reasonable. Very few would increase consumption if price dropped (Amsterdam 5%; San Francisco 13%), but slightly more would reduce consumption if price became “much more expensive” (Amsterdam 37%; San Francisco 39%). Suggests price is somewhat elastic but appears to be price-inelastic for experienced users	
		Quality	**Potency:** Majority of participants had a preference for strength (91% Amsterdam; 99% San Francisco), Amsterdam sample mostly preferred lower potency; San Francisco sample mostly preferred higher potency	
		Other	**Source:** Half of San Francisco sample obtained cannabis through friends who knew dealers; majority of Amsterdam sample (7 out of 8) purchased from regulated shops **Accessibility:** San Francisco had longer average search times	
Riley, 2020	Primary survey analysis	Price	**Price point elasticity:** − 0.501. The price for medium and high quality cannabis appears to be more elastic than low quality **Quantity discount:** A 1% increase in quantity had a 0.436% decrease in the price	
		Quality	**Quality:** If price is held constant across qualities the demand for medium quality increases by 35.5% and high quality by 81.3%	
Shi, 2019	Discrete choice experiment	Price	**Price (WTP):** Price was the most important attribute measured for non-medicinal and dual users. Medical users had higher WTP for more potent CBD products. Non-medicinal and medicinal dual users had the greatest WTP for higher THC potency products Note: There are preference heterogeneities identified by reason of use. THC potency was not as important for medical users as it was for non-medicinal cannabis users or dual users	
		Quality	**Potency:** There was a preference for higher concentrations of THC and CBD. CBD potency was the most important attribute measured for medicinal users	
		Packaging	**Warning message:** Both graphic warnings on drugged driving and text warning messages were positively received by users and nonusers, while FDA disapproval disclaimers were negatively received	
Shukla, 2003	Interviews	Price	**Cost:** Some participants indicated that insufficient disposable income would lead them to use less or none at all Note: This was a large dissertation in illegal drug desistance, portions that are relevant to this study were limited to some qualitative interviews	
		Other	**Safety:** There are risks associated with purchasing from the illicit market including dealing with strangers or going in drug houses, risk of being caught/arrested, associated with going to drug houses, risks with product uncertainty **Availability:** Individuals were generally not interested/willing to go to extreme measures to purchase cannabis when it was easily available	
Smart, 2017	Analysis of admin data	Price	**Quantity discount:** For each 10% increase in quantity, there was a 0.62% reduction in unit price. Note purchases of < 5 g accounted for ~ 75% of all transactions	
Wadsworth, 2019	Primary survey analysis	Price	**Quantity discount:** For each 10% increase in quantity, there was a 2.0% reduction in unit price **Price:** Compared to purchasing from a family member or friend, purchasing from an illicit dealer, licensed producer, and online/mail order was associated with a higher price per gram, at a rate of 16.1%, 33.5%, and 23.7% respectively	
		Other	**Source:** Most common sources were family member/friends (53.0%); illicit street dealers (51.7%)	
Williams, 2004	Secondary analysis if survey data	Price	**Price elasticity:** Both the demand and prevalence of use is responsive to change in price. Heterogeneity exists between age groups with youth participation being more price sensitive than older age groups. There was no significant difference found for the demand of cannabis between genders for those aged below 25 years old. However, females aged 25 years or older were more sensitive to price changes compared to males of the same age	
		Other	**Legal status:** Decriminalization is associated with a higher prevalence of use among males over 25 years of age. No indication that decriminalization significantly increases prevalence of use by either young persons (male or female), or increases the frequency of use among cannabis users	

With respect to price elasticity, demand was inelastic in most cases; however, some studies noted heterogeneity with respect to population and timeframe. Reinarman et al. (2009) found that the price was inelastic for experienced users and more elastic for novice users, while Williams et al. (2004) found youth to be more price sensitive than older age groups. Hansen et al. (2017) found price to be elastic in the two weeks before and after a price change as a result of a tax reform in Washington State. Finally, Riley et al. (2020) found differences in elasticity based on quality with medium and high-quality cannabis having a greater price elasticity. Davis et al. (2016) found a significant difference in the price people would pay per gram with high-quality cannabis retailing for an average of $13.77 per gram and low-quality cannabis at an average of $5.63 per gram, as per crowd sourced price data. Ben Lakhdar et al. (2016) was the only study that found price to be elastic consistently; however, this study only examined short term elasticity among regular consumers.

Five studies examined quantity discounts (Ben Lakhdar et al., 2016; Caulkins and Pacula, 2006; Riley et al., 2020; Smart et al., 2017; Wadsworth et al., 2019), and all studies found that price decreased with an increase in quantity purchased. Ben Lakhdar et al. (2019) found however that the degree of discount differed by geographic region, with larger cities offering cannabis at lower prices per gram.

One study explored the relative importance of price in purchase decisions. This discrete choice experiment found that price was an important factor in purchase decisions for all users. It was the most important attribute considered for non-medicinal and dual (non-medicinal and medicinal) consumers; however, price was not as important as CBD content for medicinal users (Shi et al., 2019).

Three studies looked at differences in price by source (Capler et al., 2017; Reed et al., 2020; Wadsworth et al., 2019). Wadsworth et al. (2019) found that compared to purchasing from a family member or friend, purchasing from an illicit dealer, licensed producer, and online/mail order was associated with a higher price per gram, at a rate of 16.1%, 33.5%, and 23.7% respectively. Capler et al. (2017) reported on satisfaction with various sources in terms of price. People were most satisfied with the price from growers, self-producers and Health Canada, somewhat satisfied with friends and dispensaries, and not satisfied with the price through street dealers. Reed et al. (2020) noted that some consumers shopped around based on new customer specials.

### Non-price-related factors

Many non-price factors were explored in the included studies. These factors have been grouped into the following broad categories: (1) quality (*n* = 11) (Boehnke et al., 2019; Capler et al., 2017; Chait and Burke, 1994; Cole et al., 2008; Gilbert and DiVerdi, 2018; Goudie et al., 2007; Halcoussis et al., 2017; Reinarman, 2009; Riley et al., 2020; Shi et al., 2019; Vincent et al., 2017), (2) route of administration (*n* = 3) (Aston et al., 2019; Boehnke et al., 2019; Capler et al., 2017), (3) product recommendations (*n* = 1) (Boehnke et al., 2019), (4) packaging (*n* = 2) (Goodman et al., 2019; Shi et al., 2019), and other (*n* = 6) (Table 4) (Boehnke et al., 2019; Capler et al., 2017; Reed et al., 2020; Reinarman, 2009; Shukla, 2003; Wadsworth et al., 2019).

#### Quality

Within the eleven studies that examined perceived cannabis quality, several different components of quality were explored. These include (1) demand elasticity based on perceived quality (*n* = 5) (Cole et al., 2008; Goudie et al., 2007; Halcoussis et al., 2017; Riley et al., 2020; Vincent et al., 2017), (2) product potency, strain (*n* = 4) (Boehnke et al., 2019; Chait and Burke, 1994; Reinarman, 2009; Shi et al., 2019), (3) aroma and visual appeal (*n* = 2) (Boehnke et al., 2019; Gilbert and DiVerdi, 2018), and quality by source (*n* = 1) (Capler et al., 2017).

##### Impact of quality on demand elasticity

Five studies looked at the impact of perceived cannabis product quality on demand (Cole et al., 2008; Goudie et al., 2007; Halcoussis et al., 2017; Riley et al., 2020; Vincent et al., 2017). Halcoussis et al. (2017) found that low and high-quality cannabis had a positive demand elasticity compared to medium-quality when price was held constant. Somewhat conversely, Riley et al. (2020) found that medium and high-quality cannabis was purchased in greater quantity than a low-quality product when price was constant. Three studies used the MPT approach to measure different types of elasticity with respect to perceived quality. The first looked at demand elasticity over different levels of quality (Cole et al., 2008) and found demand to be elastic (elasticity = − 1.31). A second study used the MPT approach but measured income elasticity over different levels of quality (Goudie et al., 2007). Demand was income inelastic for low and average quality cannabis, but income elastic for good quality cannabis. Finally, a third study measured price elasticity over different quality grades (Vincent et al., 2017); they found price elasticity to increase with increasing quality grade.

##### Potency and strain

Overall, studies found that people generally had a preference regarding THC and CBD potency and that most preferred higher concentrations. However, there was some heterogeneity depending on reason for use, geography, and gender. Two studies found that medicinal users preferred cannabis with more CBD and less THC compared to dual users who preferred higher levels of both THC and CBD (Boehnke et al., 2019; Shi et al., 2019). Although both studies found that cannabinoid content played the biggest role in determining the cannabis product selected, dual users seemed to place more value on THC concentration, while medical users placed greater value on CBD concentration. Non-medicinal users preferred more potent cannabis (Chait and Burke, 1994; Reinarman, 2009; Shi et al., 2019). With respect to gender, Boehnke et al. (2019) found that men preferred cannabis with both high THC and high CBD, while women preferred cannabis that had a low THC to high CBD ratio. Additionally, men were more likely consider the cannabinoid content when selecting a cannabis product.

One study comparing preferences between Amsterdam and San Francisco found that people from Amsterdam preferred mild and moderate-strength cannabis, while in San Francisco, they preferred strong and very strong cannabis (Reinarman, 2009). This study also found that approximately two thirds of people would use less cannabis than normal if they were using strong or very strong cannabis (Reinarman, 2009).

In the study by Boehnke et al. (2019), about half of the participants took into consideration the cannabis strain and described effects when deciding on what product to purchase. About two thirds preferred indica/sativa hybrid strains, about one quarter preferred indica strains, and 10% preferred sativa strains. Strain was more important to male users, dual users, and experienced users. Described effects were more important to dual and experienced users, but there was no difference between genders.

##### Aroma and visual appeal

Gilbert and DiVerdi (2018) found that respondents were interested in smoking cannabis with citrus/sweet/lemon/pungent aromas and that these were also perceived as more expensive and potent compared to earthy/herbal/woody aromas. The price and experimentally determined level of THC of the strains, however, did not show any relationship with that of the consumers’ perception. Boehnke et al. (2019) broke down their survey findings by reason for use, gender, and experienced versus novice users. They found that smell was of greater importance to people who consumed for both medicinal and non-medicinal purposes compared to using solely for medicinal reasons, medicinal users alone, males, and more experienced users.

Boehnke et al. (2019) was the only study that explored visual appeal. Overall, visual appeal was important to 26.3% of users and was more relevant to male users, dual users, and experienced users.

##### Quality by source

Only one study rated cannabis quality by source (Capler et al., 2017). Participants were asked to rank various cannabis characteristics based on the source. Sources by best to worst quality were dispensary, grower, self-produce, friend, street, and, finally, Health Canada.

#### Route of administration

Two surveys (Boehnke et al., 2019; Capler et al., 2017) and a qualitative study (Aston et al. 2019) looked at preferred administration route. Smoking was the preferred route at 59%; however, they found that vaporizing was the most common second choice at 29%, with about one quarter preferring oral products, followed by tinctures (13.7%), edibles (12.2%), and topical applications (4.1%) (Boehnke et al., 2019). Preferences differed by reason for use, gender, and experience. Medicinally, preferences were more scattered with one quarter preferring smoking, another quarter vaporization, less than one fifth tinctures, and about 15% edibles. Men ranked smoking and vaporizing as their preferred methods, while a higher proportion of women preferred topical and tinctures. Novice users preferred vaporizing (34.8%), followed by smoking (26.1%), tinctures (18.5%), and edibles (14.2%), while experienced users preferred smoking (47.2%), followed by vaporizing (25.6%), edibles (11.1%), and tinctures (10.9%).

Aston et al. (2019) explored medicinal cannabis users’ preferences for vaporization in more detail. Medical consumers liked the flexibility that vaporizing allowed them for dosing and timing and also found the vaping devices to be portable and discreet.

Capler et al. (2017) compared preferences for route based on the source of cannabis. They found that preference did not differ for users who acquired cannabis through dispensaries versus those who acquired from other means, including private company under contract with Health Canada, self-production, other producer, friend or acquaintance, or street dealer.

#### Product recommendations

Another factor that influenced consumer choice was product recommendations by dispensary employees and/or friends. Boehnke et al. (2019) found in their survey that, collectively, over half of medicinal and dual users credited dispensary employees in assisting them selecting a cannabis product, while under one quarter attributed recommendations from friends. A larger proportion of medicinal users, however, relied on recommendations from dispensary employees, and a large proportion of dual users relied on recommendations from friends. Experienced users were more likely to rely on recommendations from a friend, while novice users were more likely to rely on recommendations from a dispensary employee. There was no difference in preferred recommendation source between men and women.

#### Packaging

Goodman et al. (2019) determined in their survey that fully branded products were more appealing than those with either plain packaging or brand logo only. This study also found that for warning messages in general, participants ranked packages without warning messages more appealing than packages with warning messages. With respect to packaging appeal by product type, they found that edible gummies were the most appealing product, followed by pre-rolled joints, and then cannabis oil. Additionally, edible gummies and pre-rolled joints were rated to be significantly more appealing and more likely to be youth oriented when in fully branded packaging, compared to plain packaging or brand logo only packaging. However, the influence of product packaging on appeal tended to decrease with age.

Shi et al. (2019) found that cannabis consumers preferred text warning displays instead of graphic warnings in a discrete choice experiment. There was some preference heterogeneity between user types with medicinal users preferring warning displays in text, recreational non-medicinal users in graphic displays, and dual users preferred the FDA disclaimer in addition to graphic warning displays.

#### Other

Other attributes of choice that were explored include source (Reinarman, 2009; Wadsworth et al., 2019), product name (Boehnke et al., 2019), safety (Capler et al., 2017; Shukla, 2003), availability (Capler et al., 2017; Shukla, 2003), efficiency (Capler et al., 2017), feeling respected (Capler et al., 2017), accessibility (Reinarman, 2009), and delivery (Aston, 2019). For details on findings, refer to Table 4.

## Discussion

This systematic review sought to examine attributes that influenced cannabis consumers’ purchasing decisions. While price was the most researched attribute, other attributes like characteristics of quality, packaging, route of administration, and product recommendations also influenced purchase decisions. Media reports often claim that attributes such as high price, poor quality, limited supply, distance to stores, and inconvenient packaging of legal cannabis products are reasons why consumers continue to purchase from illegal sources (CBC News, 2019; Cecco, 2019; Esfandiari, 2019; Geraghty, 2019; Johnson, Glen et al., 2019; Lamers, 2019; Mazur, 2019; Tunney, 2019a; Turvill, 2020; Williams, 2019). However, as cannabis legalization is relatively recent, the number of studies which have explored these attributes is limited. In general, there is a dearth of evidence to support understanding of the role that any attributes play on cannabis consumer choice, outside of price.

The attribute of price constituted a majority of the reviewed literature. These studies were conducted mostly in populations where cannabis had not been legalized for non-medical use, while others were in populations where cannabis status was legalized, unknown, or mixed. Legalization creates a shift in the demand curve and therefore, it is important to consider this aspect when interpreting demand functions. It is also important to recognize that there is considerable heterogeneity in how the values for elasticity are derived across MPT studies. Some studies examine revealed choices by looking at transaction records, while others with examine stated choices using hypothetical scenarios. The prices and units of cannabis included varied from puffs to whole joints or grams of cannabis. Instructional vignettes and choice parameters used to describe the purchase decision varies greatly across studies which can impact demand (Aston and Meshesha, 2020). Finally, included studies used a variety of different demand equations; therefore, comparison of demand predictors cannot be compared directly to one another. The purpose of this study was not to examine the impact of price in detail. For a more in-depth interpretation of price and price elasticity measured through using MPT design, Aston et al. (2019) provides a comprehensive review.

When consumers were faced with a choice of different sources of cannabis offering the same product at different prices, people chose the product at the lower cost as shown in the study by Shi et al. (2019). In Canada, cannabis purchased from Health Canada licensed producers was associated with the greatest price per gram out of the examined sources, being over double the price per gram of illicit sources (Wadsworth et al., 2019). Quantity discount might also explain the large difference in price per gram between legal and illegal cannabis. It appears that the effect of quantity discount and a general lower price per gram of illegal cannabis may offer an explanation as to why the illicit market continues to thrive despite cannabis legalization.

Packaging also appeared to influence product selection, however studies that explored packaging did so through hypothetical questionnaires and focused solely on branding and warning messages (Shi et al., 2019; Wadsworth et al., 2019) and did not investigate legal cannabis’s oversized and wasteful packaging as described in media reports (Lamers, 2019). Given public pressure to be more environmentally conscious, excess packaging may have the potential to influence where consumers purchase cannabis. No studies have looked at the impact of packaging on real purchase decisions.

Media reports claim that legal cannabis is of lower quality than illegal cannabis (Turvill, 2020). However, there is insufficient evidence to support this claim as quality was either insufficiently defined or not examined in the studies reviewed here. Quality could be interpreted as any combination of label accuracy, potency, presence of contaminants or pesticides, curing process, ability to give desired effect, size, visual properties, and aroma (“How to buy good weed,” 2020). More research is needed to explore cannabis quality and how that is defined by consumers. One aspect of quality that perhaps does provide some insight into the strong illegal market is the higher potency of cannabis available on the illicit market (Mahamad et al., 2020). Generally speaking, medicinal users preferred high CBD content, while dual and non-medicinal users preferred high THC content (Shi et al., 2019).

Exploring gender differences that influence purchase decisions is an important consideration given that that cannabis use was more prevalent among males than females (CCSA, 2019). There also appeared to be sex-based physiological, behavioral, and neurobiological differences in cannabinoid effects, which may play a role in product selection (Fattore and Fratta, 2010). Although many of the studies examined included male and female participants, there were only a few areas where gender preferences were highlighted. Men tended to choose products with higher potency and preferred smoking or vaping, as compared to women who chose lower potency products and preferred topicals or tinctures (Boehnke et al., 2019). Men also tended to consider strains and the smell of the product when selecting cannabis to purchase (Boehnke et al., 2019). However, it is unknown if there are gender differences when considering other attributes in product selection, such as price, quality, packaging, and product recommendations. A recent scoping review reported on how gender norms influence patterns of cannabis use (Hemsing and Greaves, 2020). Further research on gender differences when choosing cannabis products is needed.

The goal of cannabis legalization in many jurisdictions is to protect public health through safety and quality regulations (ACT Government, 2020; Spithoff et al., 2015). However, cannabis is still purchased from the illegal market (Canada, 2020; George-Cosh, 2019), so a better understanding of the attributes that people consider when purchasing products will help inform the reasons for choosing between the illegal and legal markets. This study has provided a better understanding of these attributes; however, it also highlights that there are significant gaps in our knowledge in this area. A more thorough knowledge of cannabis consumer purchasing preferences can help policy makers refine the existing policies that will protect public health and safety while meeting the needs of consumers.

### Limitations

There were several limitations in this systematic review. Given cannabis legalization is relatively recent and restricted to a few countries, the literature regarding this topic is limited and the number of studies exploring each attribute is scarce, especially in a post-legalization context. Although price was the most common attributed examined, many of the studies that examined aspects of price did so using distinct methodologies and data sources that should not be considered together. In studies that captured data on purchase history through a survey, prices were often exaggerated or recalled incorrectly, whereas administrative data captured actual purchase behavior. Studies using the MPT design captured data in an experimental setting, and the effect of price on purchases were considered more objective despite being a hypothetical measure.

Outside of price, it is difficult to draw clear conclusions on the influence of other attributes on purchase decisions. Many studies focused on only one, or a small number of factors, and therefore, very little is known about the relative importance of each. There was insufficient data on many of the attributes, including aroma/taste, described effects, product recommendations, provenance, product strain, stigma, product safety, and personal safety, thus limiting the ability to make any estimate on the degree to which these attributes influence choice. Heterogeneity among the study methods and samples also makes it difficult to generalize many of the findings. Several studies had very narrow inclusion criteria and were therefore only representative of that subset of the population, or the sample size was very small. For several of the studies that met our inclusion criteria, choice attributes were often not the primary outcome examined and therefore lacked detail regarding those choice attributes.

Preferences for cannabis products likely differ across consumers based on frequency of use. There were no studies that broke down findings based on consumer use frequency. Qualitative methods were used in only three studies, which limits the depth of understanding especially around non-price attributes. There was also a lack of youth perspectives when making purchase decisions and only a few studies identified gender influences on product choice.

Finally, the literature to date has mainly focused on choices for dried flower cannabis. However, attributes of purchase choices likely differ across product types. For example, visual appeal may be less important for a cannabis beverage purchase compared to dried flower. There is currently no research evidence that helps us to appreciate heterogeneity in choice behavior by product type or route of administration.

While this study is a thorough review of the available literature on consumer preferences for cannabis products, the limitations noted above prevent us from drawing any specific conclusions based on the data.

### Future research

Future research is needed to develop a more thorough understanding of the non-price related attributes that people use when choosing cannabis products as well as the relationship between these various attributes. Factors cited by the media, such as distance to licensed stores, cannabis supply, product moisture, and bulky and wasteful packaging, lack evidence and remains to be studied. Quality of the product is poorly studied and has varied meanings, so research is needed to determine what quality means to people and how it influences purchase decisions. With the increasing use of cannabis use among youth (Canada, 2019), it will be important to explore the factors for their choices. Finally, an appreciation for potential heterogeneity among choices based on consumer characteristics (e.g., gender, reason for cannabis use, frequency of use) as well as type of cannabis product (e.g., dried flower, oil, edible) is needed.

## Conclusions

This systematic review presents a summary of findings from current literature regarding attributes of choice when consumers purchase cannabis products. The majority of studies focused on price-related attributes whereas three studies contributed a large proportion of findings for non-price attributes. Demand is generally inelastic with respect to price, but the degree of elasticity varies by age, gender, and experience with cannabis. Preferences were greater for products with higher potency of either THC or CBD, but this also changed based on reason for use and gender. There is insufficient evidence to understand the true impact of other attributes on the choices of cannabis consumers and the relationship between attributes. Going forward, additional research will support a more thorough understanding of these attributes, which can offer a better explanation of consumers’ thoughts and opinions. This information will be useful for helping policy makers refine the existing policies to better support public health and safety and meet consumer needs. It can also offer insight for countries looking to legalize cannabis for either medicinal or non-medicinal use.

## Supplementary Information

**Additional file 1.**

## Data Availability

Complete search strategy and detailed study tables are included in the supplementary appendix.

## Abbreviations

CBD: Cannabidiol
DACU: Driving after cannabis use
MMAT: Mixed Methods Appraisal Tool
MPT: Marijuana Purchase Task
THC: Tetrahydrocannabinol
