# Supplementary material for: Characteristics that influence purchase choice for cannabis products: a systematic review
Source: J Cannabis Res. 2022 Feb 1;4:9. doi: 10.1186/s42238-022-00117-0 (PMC8805380; doi:10.1186/s42238-022-00117-0)
Supplement: Supplementary file 1 — Additional file 1. [file 42238_2022_117_MOESM1_ESM.docx]

# **Supplementary Material**

**Search Strategy**

**Database Retrieval**

ABI/INFORM (via ProQuest) - 321 results

ASSIA (via ProQuest) - 349 results

Business Source Complete (via EBSCOhost) - 139 results

EMBASE (via Embase.com) - 2337 results

IBSS (via Proquest) - 231 results

Medline (via Ovid) - 1385 results

PsycINFO (via EBSCOhost) - 1191 results

SocINDEX (via EBSCOhost) - 472 results

Sociological Abstracts (via ProQuest) - 360 results

Scopus - 1625 results

**Search Strategies**

**Database: Ovid MEDLINE(R) and Epub Ahead of Print, In-Process & Other Non-Indexed** Citations and Daily <1946 to April 10, 2020>

--------------------------------------------------------------------------------

1 marijuana smoking/ (4709)

2 "marijuana use"/ (780)

3 ((marijuana or marihuana or cannabis or bhang or charas or ganja or hash or hashish or pot or dope or weed or dab or dabs or kief or shatter or budder or CBD or THC or cannabidiol) adj3 use*).ti,ab. (15584)

4 ((marijuana or marihuana or cannabis or bhang or charas or ganja or hash or hashish or pot or dope or weed or dab or dabs or kief or shatter or budder) adj2 smok*).ti,ab. (2306)

5 ((marijuana or marihuana or cannabis or bhang or charas or ganja or hash or hashish or pot or dope or weed or dab or dabs or kief or shatter or budder or CBD or THC or cannabidiol) adj oil*).ti,ab. (148)

6 ((marijuana or marihuana or cannabis or bhang or charas or ganja or hash or hashish or pot or dope or weed or dab or dabs or kief or shatter or budder or CBD or THC or cannabidiol) adj edible*).ti,ab. (34)

7 ((marijuana or marihuana or cannabis or bhang or charas or ganja or hash or hashish or pot or dope or weed or dab or dabs or kief or shatter or budder or CBD or THC or cannabidiol) and consumer*).ti,ab. (465)

8 ((marijuana or marihuana or cannabis or bhang or charas or ganja or hash or hashish or pot or dope or weed or dab or dabs or kief or shatter or budder or CBD or THC or cannabidiol) and customer*).ti,ab. (27)

9 (recreational adj (cannabis or marijuana or marihuana)).ti,ab. (472)

10 or/1-9 (18492)

11 commerce/ (24544)

12 ec.fs. (419111)

13 economic*.mp. (668769)

14 exp "Costs and Cost Analysis"/ (234132)

15 cost*.mp. (692891)

16 afford*.mp. (107073)

17 (price or prices or pricing).mp. (38439)

18 quality.mp. (1166122)

19 aroma*.mp. (135983)

20 scent*.mp. (3916)

21 taste*.mp. (42113)

22 (flavor* or flavour*).mp. (22711)

23 wet*.mp. (98697)

24 dry*.mp. (188058)

25 humidity.mp. (38490)

26 potency.mp. (106034)

27 (thc adj2 level*).mp. (225)

28 (thc adj2 percent*).mp. (24)

29 (thc adj2 ratio*).mp. (62)

30 (cbd adj2 level*).mp. (64)

31 (cbd adj2 percent*).mp. (6)

32 (cbd adj2 ratio*).mp. (55)

33 (cannabidiol adj2 level*).mp. (20)

34 (cannabidiol adj2 percent*).mp. (3)

35 (cannabidiol adj2 ratio*).mp. (16)

36 cannabinoid profile*.mp. (20)

37 terpene profile*.mp. (51)

38 strain*.mp. (997408)

39 sativa.mp. (24222)

40 indica.mp. (7848)

41 hybrid.mp. (171127)

42 (product adj selection).mp. (393)

43 warning label*.mp. (885)

44 warning lable*.mp. (1)

45 warning message*.mp. (219)

46 packaging.mp. (29182)

47 legal.mp. (114252)

48 illegal.mp. (11010)

49 dispensar*.mp. (5238)

50 illicit.mp. (23988)

51 (black adj market).mp. (317)

52 dealer*.mp. (865)

53 (delivery or deliveries).mp. (578057)

54 (local adj (shop or dispensar* or business* or producer*)).mp. (247)

55 (independent adj (shop or dispensar* or business* or producer*)).mp. (40)

56 (small adj (shop or dispensar* or business* or producer*)).mp. (1096)

57 or/11-56 (4434396)

58 Patient Preference/ (8180)

59 Economics, Behavioral/ (457)

60 Choice Behavior/ (32075)

61 exp attitude/ (565150)

62 Decision Making/ (93630)

63 preference*.mp. (164608)

64 choice*.mp. (360838)

65 attitude*.mp. (417523)

66 decision*.mp. (440017)

67 perception*.mp. (429143)

68 ((economic or consumer or customer or user) and (behavior* or behaviour*)).mp. (68058)

69 practice*.mp. (1146856)

70 substitut*.mp. (370921)

71 purchase task*.mp. (163)

72 demand.mp. (159189)

73 incentiv*.mp. (35846)

74 elasticity.mp. (58038)

75 or/58-74 (3155400)

76 10 and 57 and 75 (1479)

77 Animal/ not Human/ (4656322)

78 76 not 77 (1461)

79 limit 78 to english (1385)

***************************

**Database: EMBASE via EMBASE.com**

Search run: April 11, 2020

| **#** | **Query** | **Results** |
| --- | --- | --- |
| 1 | 'cannabis'/de OR 'cannabis use'/exp OR (((marijuana OR marihuana OR cannabis OR bhang OR charas OR ganja OR hash OR hashish OR pot OR dope OR weed OR dab OR dabs OR kief OR shatter OR budder OR cbd OR thc OR cannabidiol) NEXT/2 use*):ab,ti) OR (((marijuana OR marihuana OR cannabis OR bhang OR charas OR ganja OR hash OR hashish OR pot OR dope OR weed OR dab OR dabs OR kief OR shatter OR budder) NEXT/1 smok*):ab,ti) OR (((marijuana OR marihuana OR cannabis OR bhang OR charas OR ganja OR hash OR hashish OR pot OR dope OR weed OR dab OR dabs OR kief OR shatter OR budder OR cbd OR thc OR cannabidiol) NEXT/1 oil*):ab,ti) OR (((marijuana OR marihuana OR cannabis OR bhang OR charas OR ganja OR hash OR hashish OR pot OR dope OR weed OR dab OR dabs OR kief OR shatter OR budder OR cbd OR thc OR cannabidiol) NEXT/1 edible*):ab,ti) OR (((marijuana OR marihuana OR cannabis OR bhang OR charas OR ganja OR hash OR hashish OR pot OR dope OR weed OR dab OR dabs OR kief OR shatter OR budder OR cbd OR thc OR cannabidiol) NEXT/1 consumer*):ab,ti) OR (((marijuana OR marihuana OR cannabis OR bhang OR charas OR ganja OR hash OR hashish OR pot OR dope OR weed OR dab OR dabs OR kief OR shatter OR budder OR cbd OR thc OR cannabidiol) NEXT/1 customer*):ab,ti) OR ((recreational NEXT/1 (cannabis OR marijuana OR marihuana)):ab,ti) | 45,127 |
| 2 | 'commercial phenomena'/exp OR 'economic aspect'/de OR 'cost'/de OR 'economics'/de OR economic*:ab,ti OR cost*:ab,ti OR afford*:ab,ti OR price:ab,ti OR prices:ab,ti OR pricing:ab,ti OR quality:ab,ti OR aroma*:ab,ti OR scent*:ab,ti OR taste*:ab,ti OR flavor*:ab,ti OR flavour*:ab,ti OR wet*:ab,ti OR dry*:ab,ti OR humidity:ab,ti OR potency:ab,ti OR ((thc NEXT/1 level*):ab,ti) OR ((thc NEXT/1 percent*):ab,ti) OR ((thc NEXT/1 ratio*):ab,ti) OR ((cbd NEXT/1 level*):ab,ti) OR ((cbd NEXT/1 percent*):ab,ti) OR ((cbd NEXT/1 ratio*):ab,ti) OR ((cannabidiol NEXT/1 level*):ab,ti) OR ((cannabidiol NEXT/1 percent*):ab,ti) OR ((cannabidiol NEXT/1 ratio*):ab,ti) OR 'cannabinoid profile*':ab,ti OR 'terpene profile*':ab,ti OR strain*:ab,ti OR sativa:ab,ti OR indica:ab,ti OR hybrid:ab,ti OR 'product selection':ab,ti OR 'warning label*':ab,ti OR 'warning lable*':ab,ti OR 'warning message*':ab,ti OR packaging:ab,ti OR legal:ab,ti OR illegal:ab,ti OR dispensar*:ab,ti OR illicit:ab,ti OR 'black market':ab,ti OR dealer*:ab,ti OR delivery:ab,ti OR deliveries:ab,ti OR ((local NEXT/1 (shop OR dispensar* OR business* OR producer*)):ab,ti) OR ((independent NEXT/1 (shop OR dispensar* OR business* OR producer*)):ab,ti) OR ((small NEXT/1 (shop OR dispensar* OR business* OR producer*)):ab,ti) | 4,823,645 |
| 3 | 'patient preference'/de OR 'behavioral economics'/de OR 'decision making'/de OR 'consumer attitude'/de OR 'patient attitude'/de OR 'patient satisfaction'/de OR preference*:ab,ti OR choice*:ab,ti OR attitude*:ab,ti OR decision*:ab,ti OR perception*:ab,ti OR ((economic:ab,ti OR consumer:ab,ti OR customer:ab,ti OR user:ab,ti) AND (behavior*:ab,ti OR behaviour*:ab,ti)) OR practice*:ab,ti OR substitut*:ab,ti OR 'purchase task*':ab,ti OR demand:ab,ti OR incentiv*:ab,ti OR elasticity:ab,ti | 3,164,540 |
| 4 | #1 AND #2 AND #3 | 2,533 |
| 5 | [animals]/lim NOT [humans]/lim | 5,770,355 |
| 6 | #4 NOT #5 | 2,475 |
| 7 | #6 AND [english]/lim | 2,337 |

**Database: Business Source Complete via EBSCOhost**

Search run: April 11, 2020

| **#** | **Query** | **Results** |
| --- | --- | --- |
| S1 | TI ((marijuana OR marihuana OR cannabis OR bhang OR charas OR ganja OR hash OR hashish OR pot OR dope OR weed OR dab OR dabs OR kief OR shatter OR budder OR cbd OR thc OR cannabidiol) N2 use*) OR AB ((marijuana OR marihuana OR cannabis OR bhang OR charas OR ganja OR hash OR hashish OR pot OR dope OR weed OR dab OR dabs OR kief OR shatter OR budder OR cbd OR thc OR cannabidiol) N2 use*) OR TI ((marijuana OR marihuana OR cannabis OR bhang OR charas OR ganja OR hash OR hashish OR pot OR dope OR weed OR dab OR dabs OR kief OR shatter OR budder) N1 smok*) OR AB ((marijuana OR marihuana OR cannabis OR bhang OR charas OR ganja OR hash OR hashish OR pot OR dope OR weed OR dab OR dabs OR kief OR shatter OR budder) N1 smok*) OR TI ((marijuana OR marihuana OR cannabis OR bhang OR charas OR ganja OR hash OR hashish OR pot OR dope OR weed OR dab OR dabs OR kief OR shatter OR budder OR cbd OR thc OR cannabidiol) N1 oil*) OR AB ((marijuana OR marihuana OR cannabis OR bhang OR charas OR ganja OR hash OR hashish OR pot OR dope OR weed OR dab OR dabs OR kief OR shatter OR budder OR cbd OR thc OR cannabidiol) N1 oil*) OR TI ((marijuana OR marihuana OR cannabis OR bhang OR charas OR ganja OR hash OR hashish OR pot OR dope OR weed OR dab OR dabs OR kief OR shatter OR budder OR cbd OR thc OR cannabidiol) N1 edible*) OR AB ((marijuana OR marihuana OR cannabis OR bhang OR charas OR ganja OR hash OR hashish OR pot OR dope OR weed OR dab OR dabs OR kief OR shatter OR budder OR cbd OR thc OR cannabidiol) N1 edible*) OR TI ((marijuana OR marihuana OR cannabis OR bhang OR charas OR ganja OR hash OR hashish OR pot OR dope OR weed OR dab OR dabs OR kief OR shatter OR budder OR cbd OR thc OR cannabidiol) N1 consumer*) OR AB ((marijuana OR marihuana OR cannabis OR bhang OR charas OR ganja OR hash OR hashish OR pot OR dope OR weed OR dab OR dabs OR kief OR shatter OR budder OR cbd OR thc OR cannabidiol) N1 consumer*) OR TI ((marijuana OR marihuana OR cannabis OR bhang OR charas OR ganja OR hash OR hashish OR pot OR dope OR weed OR dab OR dabs OR kief OR shatter OR budder OR cbd OR thc OR cannabidiol) N1 customer*) OR AB ((marijuana OR marihuana OR cannabis OR bhang OR charas OR ganja OR hash OR hashish OR pot OR dope OR weed OR dab OR dabs OR kief OR shatter OR budder OR cbd OR thc OR cannabidiol) N1 customer*) OR TI (recreational N1 (cannabis OR marijuana OR marihuana)) OR AB (recreational N1 (cannabis OR marijuana OR marihuana)) | 7,443 |
| S2 | TI economic* OR AB economic* OR TI cost* OR AB cost* OR TI afford* OR AB afford* OR TI price OR AB price OR TI prices OR AB prices OR TI pricing OR AB pricing OR TI quality OR AB quality OR TI aroma* OR AB aroma* OR TI scent* OR AB scent* OR TI taste* OR AB taste* OR TI flavor* OR AB flavor* OR TI flavour* OR AB flavour* OR TI wet* OR AB wet* OR TI dry* OR AB dry* OR TI humidity OR AB humidity OR TI potency OR AB potency OR TI (thc N1 level*) OR AB (thc N1 level*) OR TI (thc N1 percent*) OR AB (thc N1 percent*) OR TI (thc N1 ratio*) OR AB (thc N1 ratio*) OR TI (cbd N1 level*) OR AB (cbd N1 level*) OR TI (cbd N1 percent*) OR AB (cbd N1 percent*) OR TI (cbd N1 ratio*) OR AB (cbd N1 ratio*) OR TI (cannabidiol N1 level*) OR AB (cannabidiol N1 level*) OR TI (cannabidiol N1 percent*) OR AB (cannabidiol N1 percent*) OR TI (cannabidiol N1 ratio*) OR AB (cannabidiol N1 ratio*) OR TI "cannabinoid profile*" OR AB "cannabinoid profile*" OR TI "terpene profile*" OR AB "terpene profile*" OR TI strain* OR AB strain* OR TI sativa OR AB sativa OR TI indica OR AB indica OR TI hybrid OR AB hybrid OR TI "product selection" OR AB "product selection" OR TI "warning lable*" OR AB "warning lable*" OR TI "warning label*" OR AB "warning label*" OR TI "warning message*" OR AB "warning message*" OR TI packaging OR AB packaging OR TI legal OR AB legal OR TI illegal OR AB illegal OR TI dispensar* OR AB dispensar* OR TI illicit OR AB illicit OR TI "black market" OR AB "black market" OR TI dealer* OR AB dealer* OR TI delivery OR AB delivery OR TI deliveries OR AB deliveries OR TI (local N1 (shop OR dispensar* OR business* OR producer*)) OR AB (local N1 (shop OR dispensar* OR business* OR producer*)) OR TI (independent N1 (shop OR dispensar* OR business* OR producer*)) OR AB (independent N1 (shop OR dispensar* OR business* OR producer*)) OR TI (small N1 (shop OR dispensar* OR business* OR producer*)) OR AB (small N1 (shop OR dispensar* OR business* OR producer*)) | 6,909,686 |
| S3 | DE "CONSUMER behavior" OR DE "CONSUMER preferences" OR DE "WILLINGNESS to pay" OR DE "CONSUMER attitudes" OR DE "CUSTOMER satisfaction" OR DE "DISCRETE choice models" OR DE "DECISION making" OR TI preference* OR AB preference* OR TI choice* OR AB choice* OR TI attitude* OR AB attitude* OR TI decision* OR AB decision* OR TI perception* OR AB perception* OR TI ((economic OR consumer OR customer OR user) AND (behavior* OR behaviour*)) OR AB ((economic OR consumer OR customer OR user) AND (behavior* OR behaviour*)) OR TI practice* OR AB practice* OR TI substitut* OR AB substitut* OR TI "purchase task*" OR AB "purchase task*" OR TI demand OR AB demand OR TI incentiv* OR AB incentiv* OR TI elasticity OR AB elasticity | 4,042,977 |
| S4 | S1 AND S2 AND S3 | 139 |

**Database: PsycInfo via EBSCOhost**

Search run: April 11, 2020

| **#** | **Query** | **Results** |
| --- | --- | --- |
| #1 | DE "Cannabis" OR DE "Hashish" OR DE "Marijuana" OR DE "Marijuana Usage" OR TI ((marijuana OR marihuana OR cannabis OR bhang OR charas OR ganja OR hash OR hashish OR pot OR dope OR weed OR dab OR dabs OR kief OR shatter OR budder OR cbd OR thc OR cannabidiol) N2 use*) OR AB ((marijuana OR marihuana OR cannabis OR bhang OR charas OR ganja OR hash OR hashish OR pot OR dope OR weed OR dab OR dabs OR kief OR shatter OR budder OR cbd OR thc OR cannabidiol) N2 use*) OR TI ((marijuana OR marihuana OR cannabis OR bhang OR charas OR ganja OR hash OR hashish OR pot OR dope OR weed OR dab OR dabs OR kief OR shatter OR budder) N1 smok*) OR AB ((marijuana OR marihuana OR cannabis OR bhang OR charas OR ganja OR hash OR hashish OR pot OR dope OR weed OR dab OR dabs OR kief OR shatter OR budder) N1 smok*) OR TI ((marijuana OR marihuana OR cannabis OR bhang OR charas OR ganja OR hash OR hashish OR pot OR dope OR weed OR dab OR dabs OR kief OR shatter OR budder OR cbd OR thc OR cannabidiol) N1 oil*) OR AB ((marijuana OR marihuana OR cannabis OR bhang OR charas OR ganja OR hash OR hashish OR pot OR dope OR weed OR dab OR dabs OR kief OR shatter OR budder OR cbd OR thc OR cannabidiol) N1 oil*) OR TI ((marijuana OR marihuana OR cannabis OR bhang OR charas OR ganja OR hash OR hashish OR pot OR dope OR weed OR dab OR dabs OR kief OR shatter OR budder OR cbd OR thc OR cannabidiol) N1 edible*) OR AB ((marijuana OR marihuana OR cannabis OR bhang OR charas OR ganja OR hash OR hashish OR pot OR dope OR weed OR dab OR dabs OR kief OR shatter OR budder OR cbd OR thc OR cannabidiol) N1 edible*) OR TI ((marijuana OR marihuana OR cannabis OR bhang OR charas OR ganja OR hash OR hashish OR pot OR dope OR weed OR dab OR dabs OR kief OR shatter OR budder OR cbd OR thc OR cannabidiol) N1 consumer*) OR AB ((marijuana OR marihuana OR cannabis OR bhang OR charas OR ganja OR hash OR hashish OR pot OR dope OR weed OR dab OR dabs OR kief OR shatter OR budder OR cbd OR thc OR cannabidiol) N1 consumer*) OR TI ((marijuana OR marihuana OR cannabis OR bhang OR charas OR ganja OR hash OR hashish OR pot OR dope OR weed OR dab OR dabs OR kief OR shatter OR budder OR cbd OR thc OR cannabidiol) N1 customer*) OR AB ((marijuana OR marihuana OR cannabis OR bhang OR charas OR ganja OR hash OR hashish OR pot OR dope OR weed OR dab OR dabs OR kief OR shatter OR budder OR cbd OR thc OR cannabidiol) N1 customer*) OR TI (recreational N1 (cannabis OR marijuana OR marihuana)) OR AB (recreational N1 (cannabis OR marijuana OR marihuana)) | 16,150 |
| #2 | DE "Economics" OR DE "Costs and Cost Analysis" OR TI economic* OR AB economic* OR TI cost* OR AB cost* OR TI afford* OR AB afford* OR TI price OR AB price OR TI prices OR AB prices OR TI pricing OR AB pricing OR TI quality OR AB quality OR TI aroma* OR AB aroma* OR TI scent* OR AB scent* OR TI taste* OR AB taste* OR TI flavor* OR AB flavor* OR TI flavour* OR AB flavour* OR TI wet* OR AB wet* OR TI dry* OR AB dry* OR TI humidity OR AB humidity OR TI potency OR AB potency OR TI (thc N1 level*) OR AB (thc N1 level*) OR TI (thc N1 percent*) OR AB (thc N1 percent*) OR TI (thc N1 ratio*) OR AB (thc N1 ratio*) OR TI (cbd N1 level*) OR AB (cbd N1 level*) OR TI (cbd N1 percent*) OR AB (cbd N1 percent*) OR TI (cbd N1 ratio*) OR AB (cbd N1 ratio*) OR TI (cannabidiol N1 level*) OR AB (cannabidiol N1 level*) OR TI (cannabidiol N1 percent*) OR AB (cannabidiol N1 percent*) OR TI (cannabidiol N1 ratio*) OR AB (cannabidiol N1 ratio*) OR TI "cannabinoid profile*" OR AB "cannabinoid profile*" OR TI "terpene profile*" OR AB "terpene profile*" OR TI strain* OR AB strain* OR TI sativa OR AB sativa OR TI indica OR AB indica OR TI hybrid OR AB hybrid OR TI "product selection" OR AB "product selection" OR TI "warning lable*" OR AB "warning lable*" OR TI "warning label*" OR AB "warning label*" OR TI "warning message*" OR AB "warning message*" OR TI packaging OR AB packaging OR TI legal OR AB legal OR TI illegal OR AB illegal OR TI dispensar* OR AB dispensar* OR TI illicit OR AB illicit OR TI "black market" OR AB "black market" OR TI dealer* OR AB dealer* OR TI delivery OR AB delivery OR TI deliveries OR AB deliveries OR TI (local N1 (shop OR dispensar* OR business* OR producer*)) OR AB (local N1 (shop OR dispensar* OR business* OR producer*)) OR TI (independent N1 (shop OR dispensar* OR business* OR producer*)) OR AB (independent N1 (shop OR dispensar* OR business* OR producer*)) OR TI (small N1 (shop OR dispensar* OR business* OR producer*)) OR AB (small N1 (shop OR dispensar* OR business* OR producer*)) | 628,820 |
| #3 | DE "Behavioral Economics" OR DE "Consumer Behavior" OR DE "Choice Behavior" OR DE "Decision Making" OR TI preference* OR AB preference* OR TI choice* OR AB choice* OR TI attitude* OR AB attitude* OR TI decision* OR AB decision* OR TI perception* OR AB perception* OR TI ((economic OR consumer OR customer OR user) AND (behavior* OR behaviour*)) OR AB ((economic OR consumer OR customer OR user) AND (behavior* OR behaviour*)) OR TI practice* OR AB practice* OR TI substitut* OR AB substitut* OR TI "purchase task*" OR AB "purchase task*" OR TI demand OR AB demand OR TI incentiv* OR AB incentiv* OR TI elasticity OR AB elasticity | 1,280,321 |
| #4 | #1 AND #2 AND #3 | 1,241 |
| #5 | #4 AND Narrow by language: English | 1,191 |

**Database: SocINDEX via EBSCOhost**

Search run: April 11, 2020

| **#** | **Query** | **Results** |
| --- | --- | --- |
| S1 | DE "MARIJUANA" OR TI ((marijuana OR marihuana OR cannabis OR bhang OR charas OR ganja OR hash OR hashish OR pot OR dope OR weed OR dab OR dabs OR kief OR shatter OR budder OR cbd OR thc OR cannabidiol) N2 use*) OR AB ((marijuana OR marihuana OR cannabis OR bhang OR charas OR ganja OR hash OR hashish OR pot OR dope OR weed OR dab OR dabs OR kief OR shatter OR budder OR cbd OR thc OR cannabidiol) N2 use*) OR TI ((marijuana OR marihuana OR cannabis OR bhang OR charas OR ganja OR hash OR hashish OR pot OR dope OR weed OR dab OR dabs OR kief OR shatter OR budder) N1 smok*) OR AB ((marijuana OR marihuana OR cannabis OR bhang OR charas OR ganja OR hash OR hashish OR pot OR dope OR weed OR dab OR dabs OR kief OR shatter OR budder) N1 smok*) OR TI ((marijuana OR marihuana OR cannabis OR bhang OR charas OR ganja OR hash OR hashish OR pot OR dope OR weed OR dab OR dabs OR kief OR shatter OR budder OR cbd OR thc OR cannabidiol) N1 oil*) OR AB ((marijuana OR marihuana OR cannabis OR bhang OR charas OR ganja OR hash OR hashish OR pot OR dope OR weed OR dab OR dabs OR kief OR shatter OR budder OR cbd OR thc OR cannabidiol) N1 oil*) OR TI ((marijuana OR marihuana OR cannabis OR bhang OR charas OR ganja OR hash OR hashish OR pot OR dope OR weed OR dab OR dabs OR kief OR shatter OR budder OR cbd OR thc OR cannabidiol) N1 edible*) OR AB ((marijuana OR marihuana OR cannabis OR bhang OR charas OR ganja OR hash OR hashish OR pot OR dope OR weed OR dab OR dabs OR kief OR shatter OR budder OR cbd OR thc OR cannabidiol) N1 edible*) OR TI ((marijuana OR marihuana OR cannabis OR bhang OR charas OR ganja OR hash OR hashish OR pot OR dope OR weed OR dab OR dabs OR kief OR shatter OR budder OR cbd OR thc OR cannabidiol) N1 consumer*) OR AB ((marijuana OR marihuana OR cannabis OR bhang OR charas OR ganja OR hash OR hashish OR pot OR dope OR weed OR dab OR dabs OR kief OR shatter OR budder OR cbd OR thc OR cannabidiol) N1 consumer*) OR TI ((marijuana OR marihuana OR cannabis OR bhang OR charas OR ganja OR hash OR hashish OR pot OR dope OR weed OR dab OR dabs OR kief OR shatter OR budder OR cbd OR thc OR cannabidiol) N1 customer*) OR AB ((marijuana OR marihuana OR cannabis OR bhang OR charas OR ganja OR hash OR hashish OR pot OR dope OR weed OR dab OR dabs OR kief OR shatter OR budder OR cbd OR thc OR cannabidiol) N1 customer*) OR TI (recreational N1 (cannabis OR marijuana OR marihuana)) OR AB (recreational N1 (cannabis OR marijuana OR marihuana)) | 6,110 |
| S2 | DE "ECONOMICS" OR DE "COMMERCE" OR DE "COST" OR TI economic* OR AB economic* OR TI cost* OR AB cost* OR TI afford* OR AB afford* OR TI price OR AB price OR TI prices OR AB prices OR TI pricing OR AB pricing OR TI quality OR AB quality OR TI aroma* OR AB aroma* OR TI scent* OR AB scent* OR TI taste* OR AB taste* OR TI flavor* OR AB flavor* OR TI flavour* OR AB flavour* OR TI wet* OR AB wet* OR TI dry* OR AB dry* OR TI humidity OR AB humidity OR TI potency OR AB potency OR TI (thc N1 level*) OR AB (thc N1 level*) OR TI (thc N1 percent*) OR AB (thc N1 percent*) OR TI (thc N1 ratio*) OR AB (thc N1 ratio*) OR TI (cbd N1 level*) OR AB (cbd N1 level*) OR TI (cbd N1 percent*) OR AB (cbd N1 percent*) OR TI (cbd N1 ratio*) OR AB (cbd N1 ratio*) OR TI (cannabidiol N1 level*) OR AB (cannabidiol N1 level*) OR TI (cannabidiol N1 percent*) OR AB (cannabidiol N1 percent*) OR TI (cannabidiol N1 ratio*) OR AB (cannabidiol N1 ratio*) OR TI "cannabinoid profile*" OR AB "cannabinoid profile*" OR TI "terpene profile*" OR AB "terpene profile*" OR TI strain* OR AB strain* OR TI sativa OR AB sativa OR TI indica OR AB indica OR TI hybrid OR AB hybrid OR TI "product selection" OR AB "product selection" OR TI "warning lable*" OR AB "warning lable*" OR TI "warning label*" OR AB "warning label*" OR TI "warning message*" OR AB "warning message*" OR TI packaging OR AB packaging OR TI legal OR AB legal OR TI illegal OR AB illegal OR TI dispensar* OR AB dispensar* OR TI illicit OR AB illicit OR TI "black market" OR AB "black market" OR TI dealer* OR AB dealer* OR TI delivery OR AB delivery OR TI deliveries OR AB deliveries OR TI (local N1 (shop OR dispensar* OR business* OR producer*)) OR AB (local N1 (shop OR dispensar* OR business* OR producer*)) OR TI (independent N1 (shop OR dispensar* OR business* OR producer*)) OR AB (independent N1 (shop OR dispensar* OR business* OR producer*)) OR TI (small N1 (shop OR dispensar* OR business* OR producer*)) OR AB (small N1 (shop OR dispensar* OR business* OR producer*)) | 497,132 |
| S3 | DE "CONSUMER preferences" OR DE "CONSUMER attitudes" OR DE "CONSUMER behavior" OR DE "CONSUMER behavior research" OR DE "DECISION making" OR TI preference* OR AB preference* OR TI choice* OR AB choice* OR TI attitude* OR AB attitude* OR TI decision* OR AB decision* OR TI perception* OR AB perception* OR TI ((economic OR consumer OR customer OR user) AND (behavior* OR behaviour*)) OR AB ((economic OR consumer OR customer OR user) AND (behavior* OR behaviour*)) OR TI practice* OR AB practice* OR TI substitut* OR AB substitut* OR TI "purchase task*" OR AB "purchase task*" OR TI demand OR AB demand OR TI incentiv* OR AB incentiv* OR TI elasticity OR AB elasticity | 530,473 |
| S4 | S1 AND S2 AND S3 | 472 |

**Database: ABI/INFORM via ProQuest**

Date searched: April 11, 2020

| **#** | **Query** | **Results** |
| --- | --- | --- |
| S1 | MAINSUBJECT.EXACT("Marijuana") OR AB,TI((marijuana OR marihuana OR cannabis OR bhang OR charas OR ganja OR hash OR hashish OR pot OR dope OR weed OR dab OR dabs OR kief OR shatter OR budder OR cbd OR thc OR cannabidiol) N/2 use*) OR AB,TI((marijuana OR marihuana OR cannabis OR bhang OR charas OR ganja OR hash OR hashish OR pot OR dope OR weed OR dab OR dabs OR kief OR shatter OR budder) N/1 smok*) OR AB,TI((marijuana OR marihuana OR cannabis OR bhang OR charas OR ganja OR hash OR hashish OR pot OR dope OR weed OR dab OR dabs OR kief OR shatter OR budder OR cbd OR thc OR cannabidiol) N/1 oil*) OR AB,TI((marijuana OR marihuana OR cannabis OR bhang OR charas OR ganja OR hash OR hashish OR pot OR dope OR weed OR dab OR dabs OR kief OR shatter OR budder OR cbd OR thc OR cannabidiol) N/1 edible*) OR AB,TI((marijuana OR marihuana OR cannabis OR bhang OR charas OR ganja OR hash OR hashish OR pot OR dope OR weed OR dab OR dabs OR kief OR shatter OR budder OR cbd OR thc OR cannabidiol) N/1 consumer*) OR AB,TI((marijuana OR marihuana OR cannabis OR bhang OR charas OR ganja OR hash OR hashish OR pot OR dope OR weed OR dab OR dabs OR kief OR shatter OR budder OR cbd OR thc OR cannabidiol) N/1 customer*) OR AB,TI(recreational N/1 (cannabis OR marijuana OR marihuana)) | 7,210 |
| S2 | AB,TI(economic* OR cost* OR afford* OR price OR prices OR quality OR aroma* OR scent* OR taste* OR flavor* OR wet* OR dry* OR humidity OR potency OR (thc N/1 level*) OR (thc N/1 percent*) OR (thc N/1 ratio*) OR (cbd N/1 level*) OR (cbd N/1 percent*) OR (cbd N/1 ratio*) OR (cannabidiol N/1 level*) OR (cannabidiol N/1 percent*) OR (cannabidiol N/1 ratio*) OR "cannabinoid profile*" OR "terpene profile*" OR strain* OR sativa OR indica OR hybrid OR "product selection" OR "warning lable*" OR "warning label*" OR "warning message*" OR packaging OR legal OR illegal OR dispensar* OR illicit OR "black market" OR dealer* OR delivery OR deliveries OR (local N/1 (shop OR dispensar* OR business* OR producer*)) OR (independent N/1 (shop OR dispensar* OR business* OR producer*)) OR (small N/1 (shop OR dispensar* OR business* OR producer*))) | 3,812,236 |
| S3 | MAINSUBJECT.EXACT("Behavioral decision theory") OR MAINSUBJECT.EXACT("Brand preferences") OR MAINSUBJECT.EXACT("Consumer attitudes") OR MAINSUBJECT.EXACT("Consumer behavior") OR MAINSUBJECT.EXACT("Behavioral economics") OR MAINSUBJECT.EXACT("Decision making") OR MAINSUBJECT.EXACT("Product choice") OR AB,TI(preference* OR choice* OR attitude* OR decision* OR perception* OR ((economic OR consumer OR customer OR user) N/2 (behavior* OR behaviour*)) OR practice* OR substitut* OR "purchase task*" OR demand OR incentiv* OR elasticity) | 2,030,857 |
| S4 | 1 AND 2 AND 3 | 321 |

**Database: ASSIA via ProQuest**

Date searched: April 11, 2020

| **#** | **Query** | **Results** |
| --- | --- | --- |
| S1 | MAINSUBJECT.EXACT("Cannabis") OR AB,TI((marijuana OR marihuana OR cannabis OR bhang OR charas OR ganja OR hash OR hashish OR pot OR dope OR weed OR dab OR dabs OR kief OR shatter OR budder OR cbd OR thc OR cannabidiol) N/2 use*) OR AB,TI((marijuana OR marihuana OR cannabis OR bhang OR charas OR ganja OR hash OR hashish OR pot OR dope OR weed OR dab OR dabs OR kief OR shatter OR budder) N/1 smok*) OR AB,TI((marijuana OR marihuana OR cannabis OR bhang OR charas OR ganja OR hash OR hashish OR pot OR dope OR weed OR dab OR dabs OR kief OR shatter OR budder OR cbd OR thc OR cannabidiol) N/1 oil*) OR AB,TI((marijuana OR marihuana OR cannabis OR bhang OR charas OR ganja OR hash OR hashish OR pot OR dope OR weed OR dab OR dabs OR kief OR shatter OR budder OR cbd OR thc OR cannabidiol) N/1 edible*) OR AB,TI((marijuana OR marihuana OR cannabis OR bhang OR charas OR ganja OR hash OR hashish OR pot OR dope OR weed OR dab OR dabs OR kief OR shatter OR budder OR cbd OR thc OR cannabidiol) N/1 consumer*) OR AB,TI((marijuana OR marihuana OR cannabis OR bhang OR charas OR ganja OR hash OR hashish OR pot OR dope OR weed OR dab OR dabs OR kief OR shatter OR budder OR cbd OR thc OR cannabidiol) N/1 customer*) OR AB,TI(recreational N/1 (cannabis OR marijuana OR marihuana)) | 5,414 |
| S2 | AB,TI(economic* OR cost* OR afford* OR price OR prices OR quality OR aroma* OR scent* OR taste* OR flavor* OR wet* OR dry* OR humidity OR potency OR (thc N/1 level*) OR (thc N/1 percent*) OR (thc N/1 ratio*) OR (cbd N/1 level*) OR (cbd N/1 percent*) OR (cbd N/1 ratio*) OR (cannabidiol N/1 level*) OR (cannabidiol N/1 percent*) OR (cannabidiol N/1 ratio*) OR "cannabinoid profile*" OR "terpene profile*" OR strain* OR sativa OR indica OR hybrid OR "product selection" OR "warning lable*" OR "warning label*" OR "warning message*" OR packaging OR legal OR illegal OR dispensar* OR illicit OR "black market" OR dealer* OR delivery OR deliveries OR (local N/1 (shop OR dispensar* OR business* OR producer*)) OR (independent N/1 (shop OR dispensar* OR business* OR producer*)) OR (small N/1 (shop OR dispensar* OR business* OR producer*))) | 218,343 |
| S3 | MAINSUBJECT.EXACT("Behavioural economics") OR  MAINSUBJECT.EXACT("Consumer studies") OR MAINSUBJECT.EXACT("Consumer satisfaction") OR MAINSUBJECT.EXACT("Consumer attitudes") OR MAINSUBJECT.EXACT("Consumer based research") OR MAINSUBJECT.EXACT("Consumer behaviour") OR AB,TI(preference* OR choice* OR attitude* OR decision* OR perception* OR ((economic OR consumer OR customer OR user) N/2 (behavior* OR behaviour*)) OR practice* OR substitut* OR "purchase task*" OR demand OR incentiv* OR elasticity) | 327,219 |
| S4 | 1 AND 2 AND 3 | 349 |

**Database: IBSS via ProQuest**

Date searched: April 11, 2020

| **#** | **Query** | **Results** |
| --- | --- | --- |
| S1 | MAINSUBJECT.EXACT("Marijuana") OR AB,TI((marijuana OR marihuana OR cannabis OR bhang OR charas OR ganja OR hash OR hashish OR pot OR dope OR weed OR dab OR dabs OR kief OR shatter OR budder OR cbd OR thc OR cannabidiol) N/2 use*) OR AB,TI((marijuana OR marihuana OR cannabis OR bhang OR charas OR ganja OR hash OR hashish OR pot OR dope OR weed OR dab OR dabs OR kief OR shatter OR budder) N/1 smok*) OR AB,TI((marijuana OR marihuana OR cannabis OR bhang OR charas OR ganja OR hash OR hashish OR pot OR dope OR weed OR dab OR dabs OR kief OR shatter OR budder OR cbd OR thc OR cannabidiol) N/1 oil*) OR AB,TI((marijuana OR marihuana OR cannabis OR bhang OR charas OR ganja OR hash OR hashish OR pot OR dope OR weed OR dab OR dabs OR kief OR shatter OR budder OR cbd OR thc OR cannabidiol) N/1 edible*) OR AB,TI((marijuana OR marihuana OR cannabis OR bhang OR charas OR ganja OR hash OR hashish OR pot OR dope OR weed OR dab OR dabs OR kief OR shatter OR budder OR cbd OR thc OR cannabidiol) N/1 consumer*) OR AB,TI((marijuana OR marihuana OR cannabis OR bhang OR charas OR ganja OR hash OR hashish OR pot OR dope OR weed OR dab OR dabs OR kief OR shatter OR budder OR cbd OR thc OR cannabidiol) N/1 customer*) OR AB,TI(recreational N/1 (cannabis OR marijuana OR marihuana)) | 2,328 |
| S2 | AB,TI(economic* OR cost* OR afford* OR price OR prices OR quality OR aroma* OR scent* OR taste* OR flavor* OR wet* OR dry* OR humidity OR potency OR (thc N/1 level*) OR (thc N/1 percent*) OR (thc N/1 ratio*) OR (cbd N/1 level*) OR (cbd N/1 percent*) OR (cbd N/1 ratio*) OR (cannabidiol N/1 level*) OR (cannabidiol N/1 percent*) OR (cannabidiol N/1 ratio*) OR "cannabinoid profile*" OR "terpene profile*" OR strain* OR sativa OR indica OR hybrid OR "product selection" OR "warning lable*" OR "warning label*" OR "warning message*" OR packaging OR legal OR illegal OR dispensar* OR illicit OR "black market" OR dealer* OR delivery OR deliveries OR (local N/1 (shop OR dispensar* OR business* OR producer*)) OR (independent N/1 (shop OR dispensar* OR business* OR producer*)) OR (small N/1 (shop OR dispensar* OR business* OR producer*))) | 919,971 |
| S3 | MAINSUBJECT.EXACT("Behavioral economics") OR MAINSUBJECT.EXACT("Decision making") OR MAINSUBJECT.EXACT("Consumer attitudes") OR MAINSUBJECT.EXACT("Consumer behavior") OR AB,TI(preference* OR choice* OR attitude* OR decision* OR perception* OR ((economic OR consumer OR customer OR user) N/2 (behavior* OR behaviour*)) OR practice* OR substitut* OR "purchase task*" OR demand OR incentiv* OR elasticity) | 777,767 |
| S4 | 1 AND 2 AND 3 | 231 |

**Database: Sociological Abstracts via ProQuest**

Date searched: April 11, 2020

| **#** | **Query** | **Results** |
| --- | --- | --- |
| S1 | MAINSUBJECT.EXACT("Marijuana") OR AB,TI((marijuana OR marihuana OR cannabis OR bhang OR charas OR ganja OR hash OR hashish OR pot OR dope OR weed OR dab OR dabs OR kief OR shatter OR budder OR cbd OR thc OR cannabidiol) N/2 use*) OR AB,TI((marijuana OR marihuana OR cannabis OR bhang OR charas OR ganja OR hash OR hashish OR pot OR dope OR weed OR dab OR dabs OR kief OR shatter OR budder) N/1 smok*) OR AB,TI((marijuana OR marihuana OR cannabis OR bhang OR charas OR ganja OR hash OR hashish OR pot OR dope OR weed OR dab OR dabs OR kief OR shatter OR budder OR cbd OR thc OR cannabidiol) N/1 oil*) OR AB,TI((marijuana OR marihuana OR cannabis OR bhang OR charas OR ganja OR hash OR hashish OR pot OR dope OR weed OR dab OR dabs OR kief OR shatter OR budder OR cbd OR thc OR cannabidiol) N/1 edible*) OR AB,TI((marijuana OR marihuana OR cannabis OR bhang OR charas OR ganja OR hash OR hashish OR pot OR dope OR weed OR dab OR dabs OR kief OR shatter OR budder OR cbd OR thc OR cannabidiol) N/1 consumer*) OR AB,TI((marijuana OR marihuana OR cannabis OR bhang OR charas OR ganja OR hash OR hashish OR pot OR dope OR weed OR dab OR dabs OR kief OR shatter OR budder OR cbd OR thc OR cannabidiol) N/1 customer*) OR AB,TI(recreational N/1 (cannabis OR marijuana OR marihuana)) | 4,618 |
| S2 | AB,TI(economic* OR cost* OR afford* OR price OR prices OR quality OR aroma* OR scent* OR taste* OR flavor* OR wet* OR dry* OR humidity OR potency OR (thc N/1 level*) OR (thc N/1 percent*) OR (thc N/1 ratio*) OR (cbd N/1 level*) OR (cbd N/1 percent*) OR (cbd N/1 ratio*) OR (cannabidiol N/1 level*) OR (cannabidiol N/1 percent*) OR (cannabidiol N/1 ratio*) OR "cannabinoid profile*" OR "terpene profile*" OR strain* OR sativa OR indica OR hybrid OR "product selection" OR "warning lable*" OR "warning label*" OR "warning message*" OR packaging OR legal OR illegal OR dispensar* OR illicit OR "black market" OR dealer* OR delivery OR deliveries OR (local N/1 (shop OR dispensar* OR business* OR producer*)) OR (independent N/1 (shop OR dispensar* OR business* OR producer*)) OR (small N/1 (shop OR dispensar* OR business* OR producer*))) | 415,301 |
| S3 | MAINSUBJECT.EXACT("Consumers") OR MAINSUBJECT.EXACT("Client Satisfaction") OR MAINSUBJECT.EXACT("Decision Making") OR AB,TI(preference* OR choice* OR attitude* OR decision* OR perception* OR ((economic OR consumer OR customer OR user) N/2 (behavior* OR behaviour*)) OR practice* OR substitut* OR "purchase task*" OR demand OR incentiv* OR elasticity) | 521,696 |
| S4 | 1 AND 2 AND 3 | 360 |

**Database: Scopus**

Date searched: April 11, 2020

| **#** | **Query** | **Results** |
| --- | --- | --- |
| S1 | TITLE-ABS(((marijuana OR marihuana OR cannabis OR bhang OR charas OR ganja OR hash OR hashish OR pot OR dope OR weed OR dab OR dabs OR kief OR shatter OR budder OR cbd OR thc OR cannabidiol) W/2 use*) OR ((marijuana OR marihuana OR cannabis OR bhang OR charas OR ganja OR hash OR hashish OR pot OR dope OR weed OR dab OR dabs OR kief OR shatter OR budder) W/1 smok*) OR ((marijuana OR marihuana OR cannabis OR bhang OR charas OR ganja OR hash OR hashish OR pot OR dope OR weed OR dab OR dabs OR kief OR shatter OR budder OR cbd OR thc OR cannabidiol) W/1 oil*) OR ((marijuana OR marihuana OR cannabis OR bhang OR charas OR ganja OR hash OR hashish OR pot OR dope OR weed OR dab OR dabs OR kief OR shatter OR budder OR cbd OR thc OR cannabidiol) W/1 edible*) OR ((marijuana OR marihuana OR cannabis OR bhang OR charas OR ganja OR hash OR hashish OR pot OR dope OR weed OR dab OR dabs OR kief OR shatter OR budder OR cbd OR thc OR cannabidiol) W/1 consumer*) OR ((marijuana OR marihuana OR cannabis OR bhang OR charas OR ganja OR hash OR hashish OR pot OR dope OR weed OR dab OR dabs OR kief OR shatter OR budder OR cbd OR thc OR cannabidiol) W/1 customer*) OR (recreational W/1 (cannabis OR marijuana OR marihuana))) | 26,697 |
| S2 | TITLE-ABS(economic* OR cost* OR afford* OR price OR prices OR quality OR aroma* OR scent* OR taste* OR flavor* OR wet* OR dry* OR humidity OR potency OR (thc W/1 level*) OR (thc W/1 percent*) OR (thc W/1 ratio*) OR (cbd W/1 level*) OR (cbd W/1 percent*) OR (cbd W/1 ratio*) OR (cannabidiol W/1 level*) OR (cannabidiol W/1 percent*) OR (cannabidiol W/1 ratio*) OR "cannabinoid profile*" OR "terpene profile*" OR strain* OR sativa OR indica OR hybrid OR "product selection" OR "warning lable*" OR "warning label*" OR "warning message*" OR packaging OR legal OR illegal OR dispensar* OR illicit OR "black market" OR dealer* OR delivery OR deliveries OR (local W/1 (shop OR dispensar* OR business* OR producer*)) OR (independent W/1 (shop OR dispensar* OR business* OR producer*)) OR (small W/1 (shop OR dispensar* OR business* OR producer*))) | 11,454,645 |
| S3 | TITLE-ABS(preference* OR choice* OR attitude* OR decision* OR perception* OR ((economic OR consumer OR customer OR user) W/2 (behavior* OR behaviour*)) OR practice* OR substitut* OR "purchase task*" OR demand OR incentiv* OR elasticity) | 6,923,583 |
| S4 | #1 AND #2 AND #3 | 1,625 |

**Quality Appraisal using the MMAT Tool**

| **Authors – Year** | **Sampling Appropriate** | **Sample Representative** | **Measures Appropriate** | **Low Risk of Non-Response** | **Statistics Appropriate** |
| --- | --- | --- | --- | --- | --- |
| **Quantitative Descriptive** | | | | | |
| Amlung, 2019 | Yes | Yes | Yes | Can’t Tell | Yes |
| Amlung, 2018 | Yes | Yes | Yes | Can’t Tell | Yes |
| Aston, 2015 | Yes | Yes | Yes | Yes | Yes |
| Aston, 2016 | Yes | Yes | Yes | Yes | Yes |
| Collins, 2014 | Yes | Yes | Yes | Yes | Yes |
| Hindocha, 2017 | Yes | No | Yes | Yes | Yes |
| Patel, 2019 | Yes | Can’t Tell | Yes | Yes | Yes |
| Peters, 2017 | Yes | Can’t Tell | Yes | Can’t Tell | Yes |
| Strickland, 2017 | Yes | Can’t Tell | Yes | Can’t Tell | Yes |
| Strickland, 2019 | Yes | Can’t Tell | Yes | Yes | Yes |
| Teeters, 2019 | Yes | Yes | Yes | Can’t Tell | Yes |
| Vincent, 2017 | Yes | Yes | Yes | Can’t Tell | Yes |
| Cole, 2008 | Yes | Yes | Yes | Yes | Yes |
| Goudie, 2007 | Yes | No | Yes | Yes | Yes |
| Nisbet, 1972 | Can’t Tell | Can’t Tell | Can’t Tell | No | Can’t Tell |
| Ben Lakhdar, 2016 | Yes | Yes | Yes | Can’t Tell | Yes |
| Caulkins, 2006 | Yes | Yes | Yes | Yes | Yes |
| Davis, 2016 | Can’t Tell | Can’t Tell | Yes | No | Yes |
| Desimone, 2003 | Yes | Yes | Yes | Can’t Tell | Yes |
| Halcoussis, 2017 | Can’t Tell | Can’t Tell | Yes | No | Yes |
| Hansen, 2017 | Yes | No | Yes | Yes | Yes |
| Reinarman, 2009 | Yes | Yes | Yes | Yes | Yes |
| Riley, 2020 | Yes | No | Yes | No | Yes |
| Smart, 2017 | Yes | Yes | Yes | Yes | Yes |
| Wadsworth, 2019 | Yes | Yes | Yes | Yes | Yes |
| Boehnke, 2019 | Yes | No | Yes | No |  |
| Capler, 2017 | Can’t Tell | No | Yes | Yes | Yes |
| Chait, 1994 | No | No | No | Yes | Yes |
| Gilbert, 2018 | Yes | Can’t Tell | Yes | Yes | Yes |
| Goodman, 2019 | Yes | Yes | Yes | Yes | Yes |
| Shi, 2019 | Yes | Yes | Yes | Yes | Yes |
| Williams, 2004 | Yes | Yes | Yes | Yes | Yes |
|  | **Appropriate Approach** | **Qualitative Data Adequate** | **Findings adequately derived** | **Interpretation substantiated by data** | **Coherence between data, collection, analysis and interpretation** |
| **Qualitative** | | | | | |
| Aston, 2019 | Yes | Yes | Yes | Yes | Yes |
| Reed, 2020 | Yes | Yes | Yes | Can’ Tell | Can’t Tell |
| Shukla, 2003 | Yes | No | No | No | No |
